# Demethoxycurcumin exhibits amoebicidal activity against *Acanthamoeba triangularis* trophozoites and cysts and inhibits encystation

**DOI:** 10.1016/j.crpvbd.2026.100362

**Published:** 2026-02-16

**Authors:** Rachasak Boonhok, Wilaiwan Senghoi, Aman Tedasen, Suthinee Sangkanu, Chooi Ling Lim, Maria de Lourdes Pereira, Mohammed Rahmatullah, Polrat Wilairatana, Christophe Wiart, Karma G. Dolma, Alok K. Paul, Madhu Gupta, Md Atiar Rahman, Kingkan Bunluepuech, Shanmuga Sundara, Tooba Mahboob, Veeranoot Nissapatorn

**Affiliations:** a Department of Medical Technology, School of Allied Health Sciences, and Research Excellence Center for Innovation and Health Products (RECIHP), Walailak University, Nakhon Si Thammarat, 80160, Thailand; b School of Allied Health Sciences, Southeast Asia Water Team (SEA Water Team) and World Union for Herbal Drug Discovery (WUHeDD), Walailak University, Nakhon Si Thammarat, 80160, Thailand; c Division of Applied Biomedical Science and Biotechnology, School of Health Sciences, International Medical University, Kuala Lumpur, 57000, Malaysia; d CICECO-Aveiro Institute of Materials and Department of Medical Sciences, University of Aveiro, Aveiro, 3810-193, Portugal; e Department of Biotechnology and Genetic Engineering, University of Development Alternative, Lalmatia, Dhaka, 1209, Bangladesh; f Department of Clinical Tropical Medicine, Faculty of Tropical Medicine, Mahidol University, Rachathewee, Bangkok, 10400, Thailand; g The Institute for Tropical Biology and Conservation, University of Malaysia Sabah, Jalan UMS, Kota Kinabalu, Sabah, 88400, Malaysia; h Department of Microbiology, Sikkim Manipal Institute of Medical Sciences (SMIMS), Sikkim, 737102, India; i School of Pharmacy and Pharmacology, University of Tasmania, Hobart, Tasmania, 7001, Australia; j Department of Pharmaceutics, Delhi Pharmaceutical Sciences and Research University, New Delhi, 110017, India; k Department of Biochemistry and Molecular Biology, University of Chittagong, Chittagong, 4331, Bangladesh; l Department of Biotechnology, AVIT Chennai Campus, Vinayaka Mission's Research Foundation (VMRF), Paiyanoor, Tamil Nadu, 603104, India; m Faculty of Pharmaceutical Sciences, UCSI University, Kuala Lumpur, 56000, Malaysia; n Futuristic Science Research Center-School of Science, World Union for Herbal Drug Discovery (WUHeDD), and Research Excellence Center for Innovation and Health Products (RECIHP), Walailak University, Nakhon Si Thammarat, 80160, Thailand

**Keywords:** Demethoxycurcumin, *Acanthamoeba triangularis*, Encystation, Starvation, Autophagy

## Abstract

*Acanthamoeba* is a ubiquitous free-living protist commonly found in soil and water, with the T4 genotype responsible for most human infections. Treatment remains challenging due to the limited efficacy of current therapeutics and the parasite’s ability to form a highly resistant double-walled cyst. In this study, we investigated the anti-*Acanthamoeba* potential of demethoxycurcumin, a bioactive curcumin derivative, using *A*. *triangularis*, a clinically relevant T4 genotype species. Among the curcumin derivatives tested, demethoxycurcumin exhibited the strongest amoebicidal activity, effectively targeting both trophozoite and cyst forms. Scanning electron microscopy revealed pronounced ultrastructural damage, including loss of acanthopodia, membrane disruption, and pore formation, indicating compromised cellular integrity. Notably, demethoxycurcumin significantly inhibited encystation under starvation conditions, maintaining the parasite in the trophozoite stage. Consistently, transcriptional analysis showed that key autophagy-related genes (*Ac*ATG3, *Ac*ATG8b, *Ac*ATG12, and *Ac*ATG16) remained close to basal levels following sublethal treatment, supporting suppression of autophagy-associated encystation. Molecular docking and dynamics simulations showed that demethoxycurcumin binds stably to Vps34 and Cdc2b of *Acanthamoeba* spp., forming key hydrogen bonds with LYS40, GLU90, LEU92, ALA153, and PHE155, along with π interactions that support enzymatic regulation. Compared to curcumin, demethoxycurcumin formed fewer but significant contacts, maintaining persistent binding and moderate flexibility over 100 ns, highlighting its potential as a selective modulator of autophagy and cell cycle pathways. Complementary network pharmacology analyses identified overlapping targets between demethoxycurcumin and *A. triangularis* infection-related proteins, highlighting hub genes such as AKT1, TNF, MMP9, CDK1, and PIK3C3, and enriched pathways in autophagy, immune regulation, oxidative stress responses, and kinase-mediated signaling. Collectively, these findings suggest that demethoxycurcumin exerts anti-*Acanthamoeba* activity through coordinated disruption of autophagy and cell-cycle regulatory networks, leading to arrest and impaired encystation of *Acanthamoeba* trophozoites.

## Highlights

• Demethoxycurcumin exerted potent amoebicidal activity against *Acanthamoeba triangularis* trophozoites and cysts.
• Treatment induced acanthopodia loss, membrane disruption, and pore formation, compromising cellular integrity.
• Encystation was inhibited under starvation, maintaining amoebas in the trophozoite stage.
• Sublethal exposure suppressed autophagy-related pathways, with ATG gene expression remaining near basal levels.
• *Acanthamoeba* Vps34-mediated autophagy and Cdc2b-dependent cell-cycle regulation were implicated in trophozoite arrest.

## Graphical abstract

**Figure:**
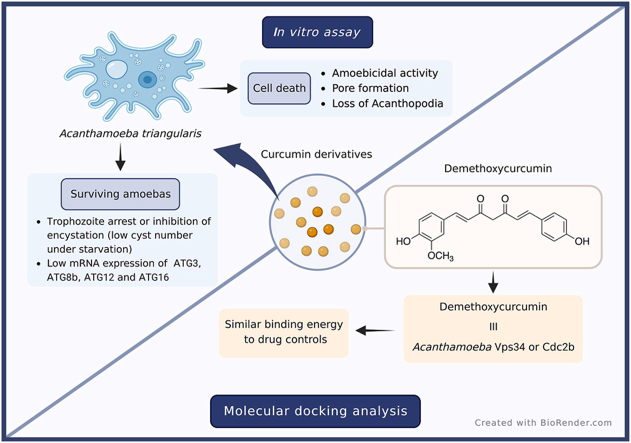

## 1 Introduction

*Acanthamoeba* is a free-living protist widely distributed in soil, water, and other environmental sources, with the T4 genotype being the most pathogenic in humans. Infection management is challenging due to the limited availability of effective drugs and the formation of double-walled cysts (Siddiqui and Khan, 2012). *Acanthamoeba castellanii*, a member of this genotype known to cause various clinical manifestations, including granulomatous amoebic encephalitis (GAE), cutaneous lesions, or chronic sinusitis, particularly in immunocompromised individuals. However, *Acanthamoeba* infections also occur in healthy individuals, especially contact lens users with poor hygiene practices. *Acanthamoeba* keratitis (AK), a severe and potentially sight-threatening ocular infection, is a significant concern in this population. Both *A. castellanii* and other T4 members, such as *A. triangularis*, have been reported to cause AK (Hasni et al., 2020). Current treatments are limited to a few medications, such as chlorhexidine and polyhexamethylene biguanide, and there is increasing concern about the development of drug resistance (Siddiqui and Khan, 2012; Chomicz et al., 2015). Thus, identifying new compounds that can inhibit cyst formation or promote amoebicidal activity is essential.

The life cycle of *Acanthamoeba* includes two distinct stages: the trophozoite, an active form that proliferates under favorable conditions, and the cyst, a dormant form that develops under environmental stress (Siddiqui and Khan, 2012). Among the proteins essential for *Acanthamoeba* growth and development are Vps34 and Cdc2b, which are conserved across eukaryotic organisms. Vps34, a class III phosphatidylinositol 3-kinase, plays a central role in regulating autophagy, supporting intracellular homeostasis and survival under stress in *Acanthamoeba* (Sakamoto et al., 2021). Cdc2b, a cyclin-dependent kinase, is crucial for cell cycle regulation, promoting parasite growth by ensuring proper cell division and facilitating encystation during unfavorable conditions (Mengue et al., 2016). Disrupting these proteins could impair essential cellular processes, such as autophagy and cell division.

*Acanthamoeba* encystation is a critical survival mechanism triggered by factors such as nutrient deprivation or chemical stress, where the trophozoite transforms into a cyst with a double-walled structure (Siddiqui and Khan, 2012). The cyst wall serves as a robust barrier, rendering *Acanthamoeba* highly resistant to therapeutic agents. Encystation in *Acanthamoeba* is closely linked to autophagy-related pathways, which allow the parasite to manage nutrient deprivation and environmental stress. Autophagy-related (Atg) proteins, including Atg3, Atg8, Atg12, and Atg16, play critical roles during autophagy and encystation. Atg3 is involved in the conjugation of Atg8 to phosphatidylethanolamine (PE), facilitating autophagosome formation (Moon et al., 2011). Atg8 contributes to autophagosome elongation and cargo recognition (Moon et al., 2011, 2013), while Atg12 and Atg16 participate in autophagosome maturation (Song et al., 2012). Understanding these molecular mechanisms provides insight into potential therapeutic targets that could disrupt the encystation.

Turmeric, derived from the rhizome of *Curcuma longa* L., is a member of the family Zingiberaceae. Rich in active components, turmeric has been used as a remedy for various human ailments for many years. Its primary bioactive compounds include curcumin, demethoxycurcumin, and bisdemethoxycurcumin, collectively known as curcuminoids (Amalraj et al., 2017), which exhibit multiple biological properties, including antimicrobial activity (Nisar et al., 2015). Recent studies have confirmed the anti-*Acanthamoeba* activity of curcumin against *A*. *triangularis*. Surviving parasites were arrested at the trophozoite stage, with diminished cyst formation and minimal upregulation of autophagy-related genes, indicating the dual role of curcumin in suppressing amoebic growth and modulating autophagy (Boonhok et al., 2022). Molecular docking has highlighted the potential of curcumin derivatives to inhibit essential enzymes and regulatory proteins involved in parasite survival, as demonstrated in a study on *Leishmania* spp. (Mohseni et al., 2022). However, the amoebicidal potential of these compounds remains underexplored.

In this study, we evaluated the amoebicidal activity of demethoxycurcumin, bisdemethoxycurcumin, and curcuminoids against *A*. *triangularis*, with particular emphasis on their effects on both trophozoite and cyst stages. We further investigated the ability of these compounds to inhibit *Acanthamoeba* encystation under nutrient-depleted (starvation) conditions. Given the close association between encystation and autophagy, we analyzed the transcriptional expression of key autophagy-related genes following demethoxycurcumin treatment to elucidate the interplay between starvation-induced autophagy and encystation. In addition, molecular docking and molecular dynamics simulations were performed to examine the interactions between demethoxycurcumin and the *Acanthamoeba* proteins Vps34 and Cdc2b, which are implicated in autophagy and cell-cycle regulation. Collectively, this integrated experimental and computational approach provides mechanistic insights into the dual potential of curcumin derivatives as amoebicidal agents and encystation inhibitors, highlighting their promise as therapeutic candidates for the management of *Acanthamoeba* infections.

## 2 Materials and methods

### 2.1 Chemicals and reagents

Chemical agents for *Acanthamoeba* culture, i.e. CaCl_2_, MgSO_4_, Na_2_HPO_4_, KH_2_PO_4_, KCl, and Tris were supplied by Sigma Aldrich (St. Louis, MO, USA); MgSO_4_·7H_2_O, CaCl_2_·2H_2_O, NaHCO_3_, was purchased from RCI Labscan (Bangkok, Thailand); (NH_4_)_2_Fe(SO_4_)_2_, glucose, and Page’s saline (PAS) powder were obtained from HiMedia (Mumbai, India). Demethoxycurcumin (CAS No. 22608-11-3), bisdemethoxycurcumin (CAS No. 33171-05-0), and curcumin (CAS No. 458-37-7, ≥ 94% curcuminoid content) were purchased from Sigma-Aldrich (St. Louis, MO, USA).

### 2.2 *Acanthamoeba* cultivation

*Acanthamoeba triangularis* WU19001, a natural isolate from natural water in Nakhon Si Thammarat, Thailand (Mitsuwan et al., 2020a), and *Acanthamoeba castellanii* ATCC50739, a clinical isolate obtained from the American Type Culture Collection (ATCC), were grown in a peptone-yeast-glucose (PYG) medium [2% (w/v) proteose peptone, 0.1% (w/v) yeast extract, 400 μM CaCl_2_, 4 mM MgSO_4_, 2.5 mM Na_2_HPO_4_, 2.5 mM KH_2_PO_4_, 50 μM (NH_4_)_2_Fe(SO_4_)_2_, 100 mM glucose], which is a full medium or nutrient-rich condition. Both species of *Acanthamoeba* were cultured at room temperature (23–25 °C) without shaking. The old medium was replaced with the fresh medium every 1–2 days until the amoeba harvesting. To prepare *Acanthamoeba* cysts, trophozoites were transferred from PYG medium to Neff’s encystment medium (0.1 M KCl, 8 mM MgSO_4_·7H_2_O, 0.4 mM CaCl_2_·2H_2_O, 1 mM NaHCO_3_, 20 mM ammediol) and cultured for 7 days without changing the medium to obtain mature cysts (Reyes-Batlle et al., 2021). Then, the cysts were collected for compound treatment.

### 2.3 Screening of amoebicidal activity and determination of minimal inhibitory concentration (MIC) of demethoxycurcumin, bisdemethoxycurcumin, and curcuminoids

Demethoxycurcumin, bisdemethoxycurcumin, and curcuminoids were prepared in 10% DMSO. To screen the amoebicidal activity against *A. triangularis* WU19001 trophozoites or cysts, the concentration of the curcumin derivatives was prepared at 1000 μg/ml (2955.52, 3243.28, and 2714.59 μM, respectively) by a broth microdilution method. Briefly, the assay was conducted in 96-well plates (SPL Life Sciences, Seoul, Korea). The compound stock solution was prepared at 2000 μg/ml (5911.04, 6486.56, 5429.18 μM, respectively) in PYG medium. Aliquots of 100 μl were transferred to each well in triplicate and mixed with 100 μl of 2 × 10^5^ trophozoites or cysts separately. Thus, the final concentration of the tested compounds was 1000 μg/ml. Plates were incubated at room temperature for 72 h. The percentage of cell viability was determined at 24, 48, and 72 h post-treatment by 0.2% trypan blue staining and quantification under an inverted microscope (Eclipse TE2000-S, Nikon, Tokyo, Japan). The relative percentage of parasite viability was defined as (Mean of treated condition/Mean of untreated control) × 100.

To determine the MIC, the microtiter broth dilution technique was performed in a 96-well plate (Mitsuwan et al., 2020a). The trophozoites or cysts of 2 × 10^5^ cells in 100 μl were prepared in the plate, treated with the compounds (starting at 1024 μg/ml with 2-fold serial dilution, 6 dilutions) at the concentrations of 1024, 512, 256, 128, 64, and 32 μg/ml, and incubated at room temperature (RT) for 24 h. The cell viability was analyzed under an inverted microscope using trypan blue staining. In our study, the MIC value denoted the lowest concentration with *Acanthamoeba* growth inhibition greater than 90%. Chlorhexidine was included as a reference anti-*Acanthamoeba* compound and used as a benchmark comparator in the assays, rather than as a fully cysticidal positive control (Tirado-Angel et al., 1996). The starting concentration was 64 μg/ml, followed by 2-fold serial dilutions to obtain 5 concentrations (64, 32, 16, 8, and 4 μg/ml). A final concentration of 1% (v/v) DMSO, corresponding to the highest solvent concentration used in the study, was applied consistently as the vehicle (negative) control, while untreated amoebae (medium only) were included as an additional baseline control. Preliminary experiments confirmed that 1% DMSO had no detectable effect on *Acanthamoeba* trophozoite or cyst viability.

### 2.4 Scanning electron microscopy (SEM)

The effect of demethoxycurcumin, bisdemethoxycurcumin, and curcuminoids on the *Acanthamoeba* trophozoites and cysts was examined separately. Methods used for parasite treatment and preparation for SEM were performed according to Sangkanu et al. (2021). In brief, *Acanthamoeba* samples were treated with each compound at its respective MIC in 24-well plates: demethoxycurcumin (1513.23 μM), bisdemethoxycurcumin (3321.12 μM), and curcuminoids (2779.74 μM). Pieces of sterile glass for parasite binding were added to the well, and the medium was gently resuspended. The plate was left at RT for 24 h. Then, the *Acanthamoeba*-coated glass slides were fixed with 2.5% glutaraldehyde and dehydrated in ethanol series (20%, 40%, 60%, 80%, 90%, and 100%). The amoeba samples were later mounted, critical-point dried, and coated with gold (Cressington 108 sputter coater, MA, USA). The amoeba surface membrane was then ready for analysis by SEM (Gemini, Oberkochen, Germany).

### 2.5 *Acanthamoeba* cyst formation under curcumin derivative treatment

A sublethal dose (0.1 × MIC) of the compounds was used for *Acanthamoeba* treatment, i.e. 147.78 μM (50 μg/ml) of demethoxycurcumin, 324.33 μM (100 μg/ml) of bisdemethoxycurcumin, and 271.46 μM (100 μg/ml) of curcuminoids. On the day of treatment, under nutrient-rich conditions (or called full condition), trophozoites of *A. triangularis* were centrifuged and placed in PYG medium, while under nutrient-depleted conditions (or starvation), trophozoites were washed 3 times with Page’s saline (PAS) buffer and placed in PAS supplemented with 5% glucose. Parasites prepared in a 96-well plate were then treated with the compounds at the final concentrations mentioned above. Starvation alone was also performed to induce *Acanthamoeba* cyst formation and used as a positive control in the assay. After 24 h treatment, the parasites were stained with trypan blue to assess parasite viability, followed by cyst formation as described by Boonhok et al. (2021). To distinguish mature cysts from trophozoites and immature cysts, samples were subjected to a 5% (v/v) SDS, and cyst quantification was performed after SDS treatment, ensuring that only SDS-resistant cysts were counted (Martín-Navarro et al., 2014). Accordingly, cysts quantified at the 24 h time-point met accepted criteria for mature *Acanthamoeba* cysts based on established SDS resistance methodology. Total parasites of at least 200 cells per condition were analyzed and cysts were quantified and represented as a percentage of cysts. The assay was done with 3 independent experiments.

### 2.6 Preparation of total RNA and cDNA synthesis

*Acanthamoeba triangularis* culture under different conditions was conducted in a 24-well plate with cell number per well of 2 × 10^5^ trophozoites. The amoebas were treated with the compounds at the concentration of 147.78 μM (50 μg/ml) demethoxycurcumin, 324.33 μM (100 μg/ml) bisdemethoxycurcumin, and 271.46 μM (100 μg/ml) curcuminoids for 24 h either under nutrient-rich or nutrient-depleted conditions and were collected at 6 and 24 h post-treatment. Preparation of total RNA and cDNA synthesis were conducted according to the methods of Boonhok et al. (2021). Then, the cDNA samples were stored at −20 °C for the qPCR experiment.

### 2.7 Validation of the PCR primers

The primers targeted *Acanthamoeba* autophagy-related genes: ATG3 (GenBank: GU270859); ATG8b (GenBank: KC524507.1); ATG12 (GenBank: HQ830265.1); ATG16 (GenBank: FJ906697); aminopeptidase (AM) (GenBank: ACA1_264610); Shwachman-Bodian-Diamond syndrome (SBDS or SB) (GenBank: ACA1_142090); cell division control protein 2b (Cdc2b or CD) (GenBank: XM_004353658.1). 18S rRNA was used as an internal control gene. The primer specificity testing was conducted before running quantitative PCR by performing conventional PCR against *A. triangularis* strain WU19001 DNA and sequencing of the PCR products (Apical Scientific Sdn. Bhd., Selangor, Malaysia).

### 2.8 Analysis of gene expression by real-time quantitative PCR

The methods used in this study were performed according to Boonhok et al. (2022). In brief, iTaq Universal SYBR Green Supermix Kit obtained from Bio-Rad (Hercules, USA) was used for the preparation of quantitative PCR reaction (qPCR). The components of the reaction were 2 × iTaq Universal SYBR Green Supermix, 200 mM of forward and reverse primers, and the cDNA samples. Then, the total volume was adjusted up to 20 μl. The StepOnePlus Real-time PCR system (Applied Biosystems, Waltham, USA) was used with the PCR cycle adjusted as follows: (i) holding stage at 95 °C for 30 s; (ii) cycling stage for 40 cycles at 95 °C for 15 s and 60 °C for 60 s; and (iii) melting curve stage at 95 °C for 15 s, 60 °C for 60 s, and 95 °C for 15 s, with a temperature increase of 0.3 °C. Calculation of a delta-delta Ct (ΔΔCt) and a relative expression was performed as described by Livak and Schmittgen (2001) using the formula for the ΔΔCt: ΔΔCt = [(Ct of treated sample GOI – Ct of treated sample housekeeper) – (Ct of untreated control GOI – Ct of untreated control housekeeper)], where GOI is the gene of interest. The formula for the relative expression of mRNA is the relative expression = 2 to the power of -ΔΔCt or 2^−ΔΔCt^. Relative gene expression was quantified using the 2^−ΔΔCt^ method and reported as fold change relative to the control condition.

### 2.9 Molecular docking study

To elucidate the interaction modes and mechanisms of action between Cdc2b and Vps34 proteins with selected bioactive compounds, molecular docking was performed. Ligand preparation included the bioactive compounds demethoxycurcumin (PubChem CID: 5469424) and curcumin (PubChem CID: 969516), along with positive control drugs, i.e. vemurafenib (PubChem CID: 42611257), MBQ-167 (PubChem CID: 129896932), GSK-F1 (PubChem CID: 57406702) and AG-1478 (2051). Structural data for all compounds were retrieved in SDF format from the PubChem database (https://pubchem.ncbi.nlm.nih.gov/). Ligands were optimized by assigning bond orders, angles, and topology, with missing and polar hydrogens added at pH 7.4, followed by conversion to PDB format and charge assignment *via* the AM1-BCC algorithm within the AMBER force field. Subsequent energy minimization and structural refinement were performed in UCSF Chimera v. 1.17.19 using steepest descent and conjugate gradient methods to ensure proper ionization correction and docking readiness.

Since experimentally resolved three-dimensional structures of Cdc2b and Vps34 proteins from *Acanthamoeba* spp. are not available in the RCSB Protein Data Bank, their structural models were retrieved from the AlphaFold Protein Structure Database (https://alphafold.ebi.ac.uk/). AlphaFold was selected as the source because of its high predictive accuracy and reliability in generating near-experimental quality protein conformations, thereby providing suitable models for subsequent molecular docking and interaction analyses. Protonation states and ion charges of Vps34 (AF-L8GS90-F1) and Cdc2b (AF-L8HG09-F1) were calculated and adjusted to physiological pH 7.4 using AutoDock Tools (ADT) v. 4.2.6, after which the refined structures were exported in both PDB and PDBQT formats.

Docking grid parameters were first defined using AutoGrid 4.2, after which molecular docking simulations were performed in AutoDock 4.2.6 employing the Lamarckian genetic algorithm with 50 GA runs and a population size of 200 to ensure robust sampling of conformational space. Positive control compounds were incorporated to provide experimentally validated binding references, thereby benchmarking the accuracy and reliability of the docking protocol. The resulting docking poses were ranked according to binding energy (kcal/mol), with the most favorable conformation identified as the one exhibiting the lowest binding energy and strongest interaction profile. Comparative analyses were then conducted between natural ligands and reference drugs to evaluate differences in binding affinity and interaction patterns. Finally, detailed examination of binding modes, hydrogen bonding, and chemical interaction networks was carried out using AutoDock 4 in conjunction with BIOVIA Discovery Studio 2025, enabling comprehensive visualization and interpretation of protein-ligand interactions (Tedasen et al., 2024).

### 2.10 Molecular dynamics simulation

Molecular dynamics (MD) simulations were employed to investigate the time-dependent behavior of the Cdc2b-demethoxycurcumin and Cdc2b-curcumin complexes, providing insights into how ligand binding influences protein flexibility, conformational stability, and interaction dynamics. Prior to simulation, each complex was carefully prepared at physiological pH 7.0 using the protein-preparation wizard: hydrogen atoms were added, bond orders were assigned, missing side chains and loops were reconstructed, hydrogen-bond networks were optimized, and water orientations were sampled to ensure structural completeness. The systems were then solvated in an orthorhombic box (10 Å × 10 Å × 10 Å) filled with TIP3P water molecules, neutralized with Na^+^ and Cl^−^ ions to a physiological concentration of 0.15 M, and parameterized for MD simulation. Production simulations were executed for 100 ns under an NPT ensemble, maintaining a constant temperature of 310 K and pressure of 1.01 bar to mimic physiological conditions. Long-range electrostatic interactions were treated using the Smooth Particle Mesh Ewald (PME) method, while solvent molecules were represented with a simple point-charge model to balance computational efficiency and accuracy. Post-simulation trajectory analyses were performed using the Simulation Interaction Diagram wizard, which included evaluation of ligand-protein contact maps, root-mean-square deviation (RMSD) profiles, root-mean-square fluctuation (RMSF) plots, and timeline interaction analyses for both ligand and protein atoms. All MD simulations and subsequent analyses were conducted using Desmond module (Schrödinger Release, 2023-2; https://www.schrodinger.com/life-science/download/release-notes/release-2023-2/), enabling a comprehensive assessment of structural stability, conformational transitions, and critical interaction hotspots throughout the simulation period. These results provided a detailed mechanistic understanding of how curcumin derivatives modulate Cdc2b protein dynamics and binding behavior (Boonsit et al., 2025).

### 2.11 Network pharmacology

To evaluate the therapeutic potential of curcumin derivatives against *Acanthamoeba* infections in humans, a comprehensive network pharmacology strategy was applied, integrating compound-target prediction, disease gene mining, and pathway enrichment analyses. Curcumin and demethoxycurcumin were retrieved from PubChem in canonical SMILES format, and their putative protein targets were predicted using SwissTargetPrediction (http://www.swisstargetprediction.ch/). Duplicate targets were removed to ensure accuracy and avoid redundancy, while a focused gene set associated with *Acanthamoeba* infection was assembled from GeneCards (https://www.genecards.org/) based on high-confidence relevance scores. The predicted targets of curcumin derivatives were then overlapped with this disease-specific gene set using the InteractiVenn tool (https://www.interactivenn.net/), thereby identifying shared nodes that represent potential therapeutic intervention points. Protein-protein interaction networks were subsequently constructed in STRING (https://string-db.org/), and functional enrichment analyses were performed using KEGG and GO pathways *via* ShinyGO v. 0.82 (http://bioinformatics.sdstate.edu/go/). These analyses highlighted critical biological processes and pathways, including PI3K/VPS34-mediated autophagy, cell-cycle regulation, oxidative stress response, and host inflammatory signaling (Chatatikun et al., 2024). Collectively, this integrated workflow provided a systems-level perspective on how curcumin derivatives exert multi-target effects, disrupt encystation, and modulate host-pathogen interactions, underscoring their promise as effective anti-amoebic therapeutic candidates.

### 2.12 Statistical data analysis

To assess the differences between the treated and untreated conditions, statistical analysis throughout the study was performed using Prism 5 software (Graphpad Prism Software, San Diego, USA). The mean ± standard deviation (SD)/standard error of the mean (SEM) together with a two-tailed unpaired Student’s t-test (∗*P* < 0.05; ∗∗*P* < 0.01; ∗∗∗*P* < 0.001) was analyzed. The results were representative of 3 independent experiments.

## 3 Results

### 3.1 Amoebicidal activity and determination of minimal inhibitory concentration (MIC) of curcumin derivatives against *A*. *triangularis*

**Fig. 1:**
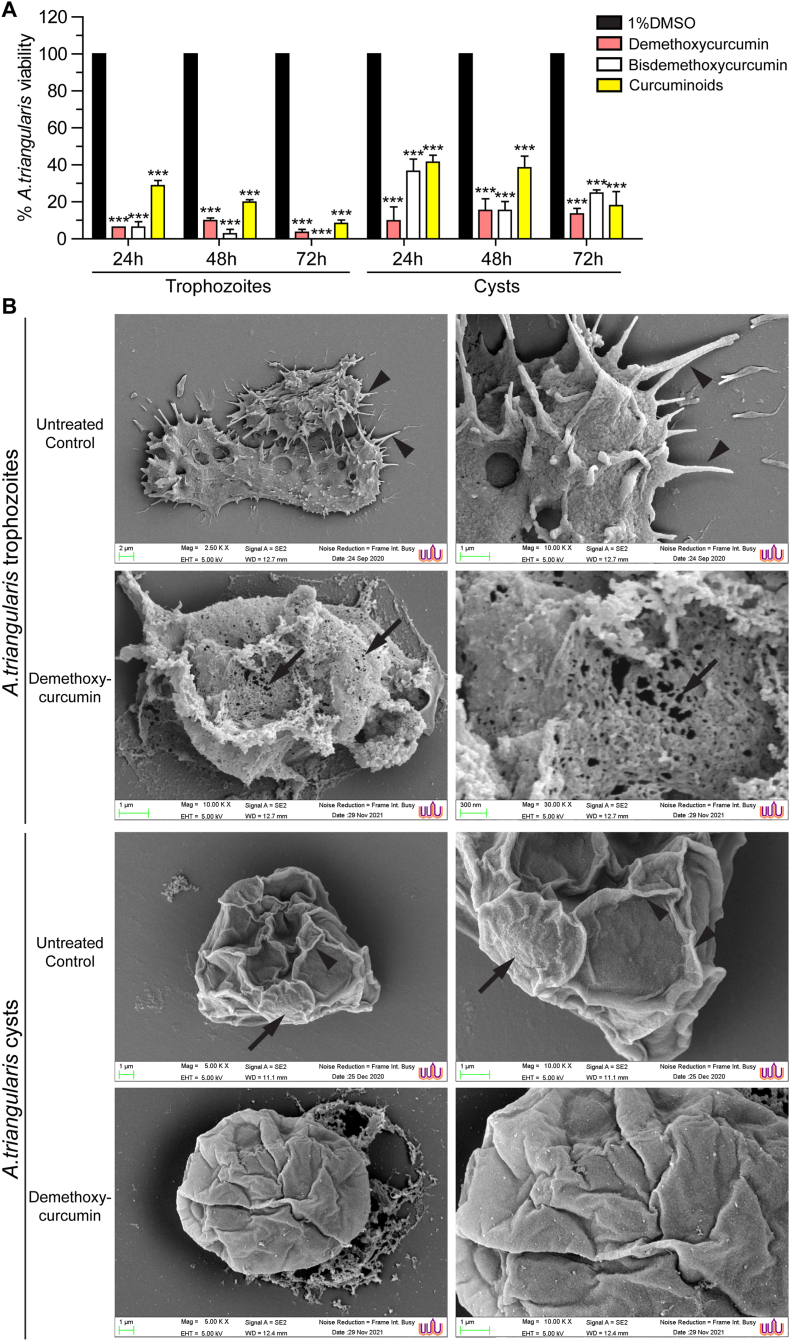
Amoebicidal activity of curcumin derivatives against *A. triangularis* trophozoites and cysts. **A** Trophozoites and cysts were treated with curcumin derivatives. The viable cells at different time points were analyzed, and represented as a percentage of *A*. *triangularis* viability. Data were obtained from 3 independent experiments. Bar graphs show the mean ± standard deviation (SD). ∗∗∗*P* < 0.001. **B** Representative images by SEM of *A*. *triangularis* treated with demethoxycurcumin at 24 h post-treatment. *Trophozoite panel*: Arrowheads indicate acanthopodia; arrows indicate porous membrane. *Cyst panel*: Arrowheads indicate venation; arrows indicate ostiole with circular pronounced edges.

To investigate the amoebicidal activity of the curcumin derivatives, the *Acanthamoeba* trophozoites and cysts were prepared and treated with the compounds at 1000 μg/ml. A 1% (v/v) DMSO (final concentration) vehicle control was included. All tested curcumin derivatives demonstrated the cidal activity to different degrees. The percentage of *A*. *triangularis* viability of both trophozoite and cyst is shown in Fig. 1A. At 24 h post-treatment, the amoebicidal activity of demethoxycurcumin and bisdemethoxycurcumin against trophozoites was greater than 90% while in curcuminoids, the cidal activity was slightly lower, 71.28%. As expected, the amoebicidal activity against the cyst form was lower compared to trophozoite stage. Nonetheless, demethoxycurcumin exhibited the highest efficacy among the tested curcumin derivatives, achieving 90.25% cidal activity at 24 h post-treatment. The cidal activity of bisdemethoxycurcumin and curcuminoids was 63.42% and 58.54%, respectively. Moreover, their amoebicidal effect persisted throughout the treatment period, from 24 to 72 h post-treatment.

The MIC was identified based on the amoebicidal activity screening results, and the data are shown in Table 1. Demethoxycurcumin demonstrated a MIC of 1513.23 μM (512 μg/ml) against *A*. *triangularis* trophozoites, while the MICs for bisdemethoxycurcumin and curcuminoids were 3321.12 μM (1024 μg/ml) and 2779.74 μM (1024 μg/ml), respectively. However, the MIC of all tested compounds against *A*. *triangularis* cysts was 3026.45 μM (1024 μg/ml), 3321.12 μM (1024 μg/ml), and 2779.74 μM (1024 μg/ml), respectively. The MIC of chlorhexidine against *A*. *triangularis* trophozoites and cysts was 15.83 μM (8 μg/ml) and 126.62 μM (64 μg/ml), respectively. Additionally, demethoxycurcumin was tested against *A*. *castellanii* ATCC 50739 trophozoites, and the MIC was found to be 3026.45 μM (1024 μg/ml). The MIC of chlorhexidine was also increased to 63.31 μM (32 μg/ml) compared to *A. triangularis* trophozoites (Supplementary Table S1).

**Table 1:** Determination of the minimal inhibitory concentration of the compounds against *A. triangularis* trophozoites and cysts at 24 h.

MIC (μM)	Trophozoites	Cysts
Demethoxycurcumin	1513.23	3026.45
Bisdemethoxycurcumin	3321.12	3321.12
Curcuminoids	2779.74	2779.74
Chlorhexidine	15.83	126.62

### 3.2 Ultrastructure of *A*. *triangularis* upon curcumin derivative treatment

To examine the structural change of trophozoites and cysts of *A. triangularis*, a SEM study was conducted. Untreated amoeba was included as a negative control and chlorhexidine-treated condition was included as a positive control for *Acanthamoeba* elimination. Upon treatment with demethoxycurcumin, bisdemethoxycurcumin, or curcuminoids, acanthopodia were absent in all treated conditions and pore formation was observed on trophozoite cell membrane. Trophozoites were still in shape under demethoxycurcumin and bisdemethoxycurcumin treatment whereas in curcuminoids-treated condition, most of the cells were rounded and some were flattened on the solid phase. The cysts of *A. triangularis* in the untreated condition clearly demonstrated key structures such as ostioles with circular or polygonal pronounced edges, venations, etc. Under compound-treated conditions, venation was no longer found on the cyst wall. Cyst swelling was observed in all treated conditions. The ostiole was absent in all groups except under curcuminoid-treated condition, where it remained visible. Representative images of demethoxycurcumin-treated condition are shown in Fig. 1B. The images for other curcumin derivatives tested are provided in Supplementary Figs. S1 and S2.

### 3.3 Cyst formation in *A*. *triangularis* under curcumin derivative treatment

**Fig. 2:**
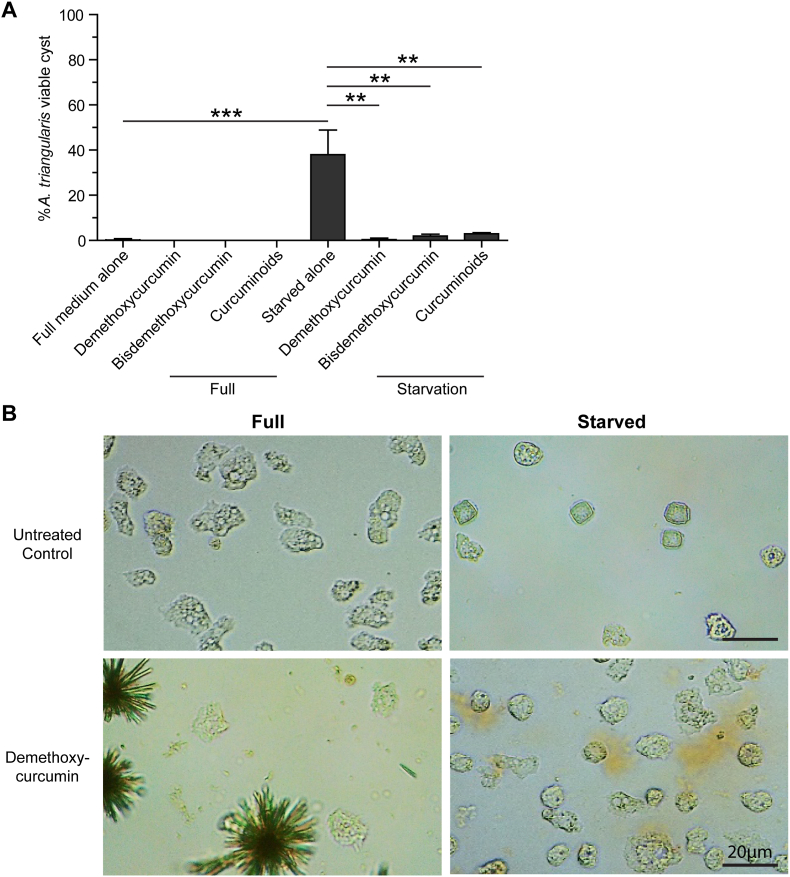
Cyst formation in *A. triangularis* under nutrient-rich and nutrient-depleted conditions. **A** Trophozoites of *A*. *triangularis* were treated with curcumin derivatives at sublethal doses for 24 h under nutrient-rich (full) or nutrient-depleted (starvation) conditions. The viable *A*. *triangularis* were quantified and represented as a percentage of the cysts. Data were obtained from 3 independent experiments. Bar graphs show the mean ± standard deviation (SD). ∗∗*P* < 0.01; ∗∗∗*P* < 0.001. **B** Representative images of the demethoxycurcumin-treated *A*. *triangularis* trophozoites under full (*left*) and starved (*right*) conditions. *Scale-bars*: 20 μm.

Demethoxycurcumin, bisdemethoxycurcumin, and curcuminoids were administered to trophozoites of *A. triangularis* for 24 h at a sublethal dose, and the survived amoebas were examined. *Acanthamoeba* cultured in full medium alone or starved medium alone was included as negative and positive control for *Acanthamoeba* cyst formation, respectively. The percentage of cysts in all treated conditions was at the basal level and similar to full medium alone (PYG medium alone). Under starved conditions, starved alone (PAS medium alone), cyst formation was induced at *c*.50%. Interestingly, in the presence of curcumin derivatives, cyst number was significantly reduced to the basal level. The percentages of cysts formed under demethoxycurcumin, bisdemethoxycurcumin, and curcuminoid treatments were 0.37%, 1.95%, and 2.96%, respectively (Fig. 2A). Representative images by inverted microscope of the untreated control and demethoxycurcumin-treated condition are shown in Fig. 2B; images for the remaining curcumin derivative-treated conditions are provided in Supplementary Fig. S3.

### 3.4 Transcriptional expression of autophagy-related genes under demethoxycurcumin treatment

**Fig. 3:**
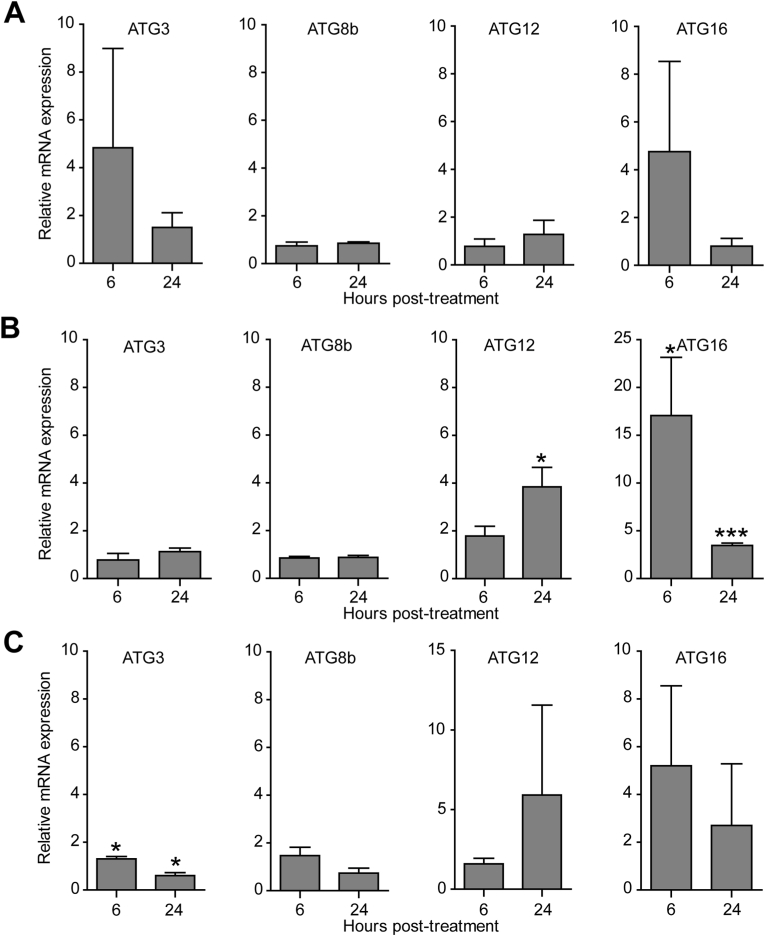
Transcriptional expression of autophagy-related genes. Trophozoites of *A*. *triangularis* were treated with demethoxycurcumin for 24 h. Total mRNA was collected at 6 and 24 h post-treatment, and qPCR was performed; 18S rRNA was used as a reference gene. Autophagy-related genes, i.e. ATG3, ATG8b, ATG12, ATG16 were analyzed. **A** Expression levels of the mRNAs under full conditions + demethoxycurcumin. **B** Expression levels of the mRNAs under starvation alone. **C** Expression levels of the mRNAs under starvation + demethoxycurcumin. Data were obtained from 3 independent experiments. Bar graphs represent the mean ± standard error of the mean (SEM). ∗*P* < 0.05; ∗∗∗*P* < 0.001.

Prior to conducting qPCR, all PCR primers (Supplementary Table S2) were validated by conventional PCR (Supplementary Fig. S4) and DNA sequencing of the PCR products was analyzed (Supplementary Table S3). Analysis of ATG3, ATG8b, ATG12, and ATG16 mRNA expression was performed. In the presence of demethoxycurcumin under the full condition, at 6 h post-treatment, the mRNA expression of ATG3 and ATG16 was slightly increased, while ATG8b and ATG12 were unchanged. However, at 24 h post-treatment, the expression of all tested ATG genes was maintained at the basal level (Fig. 3A). Under the starvation condition, the mRNA expression of ATG12 and ATG16 was significantly upregulated, whereas the expression of ATG3 and ATG8b was unchanged (Fig. 3B). However, in the presence of demethoxycurcumin, the expression of ATG16 was reduced, ATG12 showed minor fluctuations with no longer significant changes, and the expression of the other two ATG genes remained at basal levels (Fig. 3C).

The mRNA expression of other *Acanthamoeba* encystation-related genes, i.e. aminopeptidase (AM) and Shwachman-Bodian-Diamond syndrome (SB), as well as *Acanthamoeba* gene related to cell cycle, cell division control protein 2b (Cdc2b or CD), were also examined (Supplementary Fig. S5). All primer sets produced single, sharp melting peaks without evidence of non-specific amplification or primer-dimer formation, confirming the specificity of amplification for both longer and shorter qPCR targets (Supplementary Fig. S6). Under the full condition with demethoxycurcumin, the mRNA expression of aminopeptidase and Shwachman-Bodian-Diamond syndrome was slightly changed whereas the expression of Cdc2b was unchanged at both 6 and 24 h post-treatment. Under the starved condition, similar results were observed for the aminopeptidase and Shwachman-Bodian-Diamond syndrome, while the expression of Cdc2b was increased at 6 h post-treatment and returned back to the basal level at 24 h post-treatment. Taken together, in the presence of demethoxycurcumin, the expression of the tested *Acanthamoeba* encystation-related genes, including autophagy-related genes as well as the cell cycle-related gene, was regulated and maintained at the basal level.

### 3.5 Molecular docking analysis of demethoxycurcumin against Vps34 and Cdc2b proteins from *Acanthamoeba* spp

**Fig. 4:**
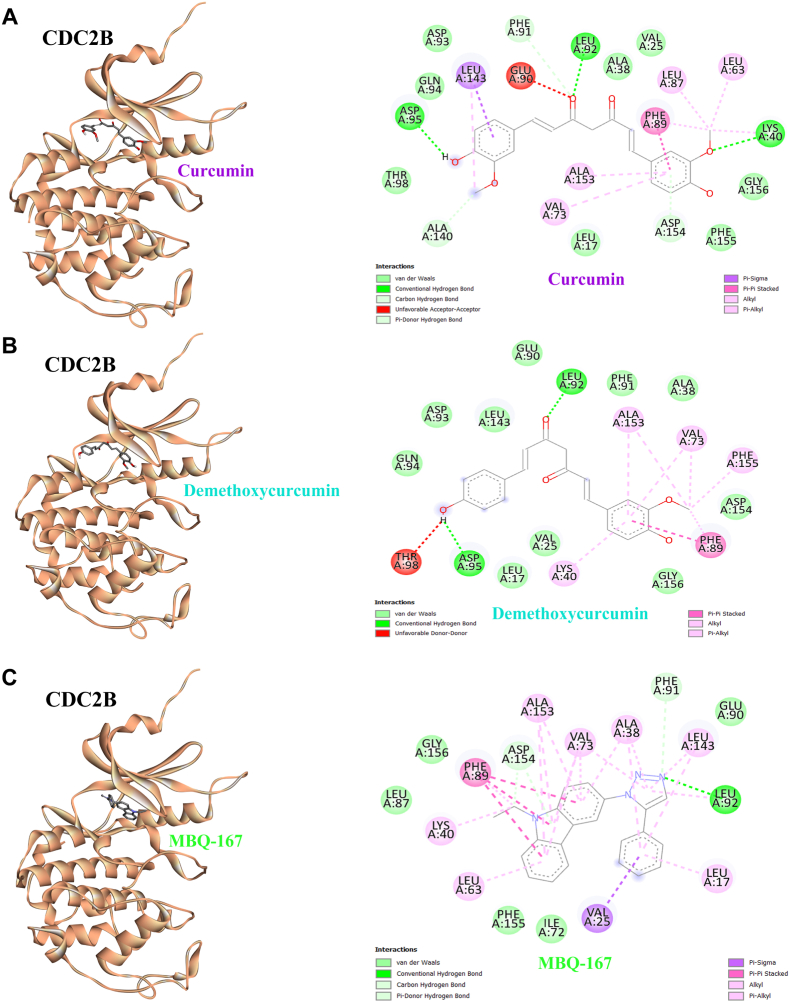
Binding modes of *A*. *triangularis* Cdc2b with curcumin, demethoxycurcumin, and MBQ-167. **A** Three-dimensional binding conformation of curcumin within the Cdc2b binding pocket (*left*) and the corresponding two-dimensional protein-ligand interaction map (*right*), illustrating key hydrogen bonds, hydrophobic contacts, and π-π interactions stabilizing the complex. **B** Binding conformation of demethoxycurcumin in complex with Cdc2b (*left*) and its two-dimensional interaction diagram (*right*), highlighting conserved and distinct residue interactions compared with curcumin. **C** Predicted binding conformation of MBQ-167 within the Cdc2b active site (*left*) and the associated interaction network (*right*), showing extensive hydrophobic and aromatic interactions with surrounding residues.

Regarding the demethoxycurcumin biological activities, it was selected for further investigation for its mechanism in the inhibition of Acanthamoeba encystation by a molecular docking study. Two target proteins were selected as candidate proteins: (i) vacuolar protein sorting 34 (Vps34), a class III phosphoinositide 3-kinase (PI3K) that plays an important role in autophagy (Boonhok et al., 2022); and (ii) cell division control protein 2b (Cdc2b), a putative regulator of the *Acanthamoeba* spp. cell cycle (Mengue et al., 2016). Against VPS34, curcumin achieved −6.26 kcal/mol (25.75 μM) with six hydrogen bonds, whereas demethoxycurcumin displayed slightly higher affinity (−6.91 kcal/mol, 8.6 μM) but only one hydrogen bond with LEU413 (Table 2 and Fig. 4A). Compared to control inhibitors such as vemurafenib, MBQ-167, and GSK-F1, which demonstrated stronger binding energies (−8.09 to −9.24 kcal/mol) and lower inhibition constants, curcumin derivatives showed moderate activity, while AG-1478 exhibited weaker binding (Table 2 and Fig. 4C). The docking analysis demonstrated that both curcumin and demethoxycurcumin exhibited favorable binding affinities toward Cdc2b of *Acanthamoeba* spp., with curcumin showing stronger binding to Cdc2b (−9.00 kcal/mol, inhibition constant 255.77 nM) through five hydrogen bonds involving residues LYS40, PHE91, LEU92, ASP95, and ASP154, while demethoxycurcumin bound Cdc2b at −8.07 kcal/mol (1.21 μM) with two hydrogen bonds to LEU92 and ASP95 (Table 2 and Fig. 4B). Curcumin also formed multiple stabilizing contacts within the Cdc2b active site, including van der Waals interactions and hydrophobic interactions, although one unfavorable donor-donor interaction was observed (Fig. 4A). Notably, demethoxycurcumin formed fewer hydrogen bonds than curcumin, yet its interactions with LEU92 and ASP95 in Cdc2b are significant, as these residues are critical for ligand recognition and enzymatic regulation, suggesting that even limited hydrogen bonding can contribute to functional modulation. Demethoxycurcumin demonstrated diverse interaction profiles, combining conventional hydrogen bonds, van der Waals forces, and multiple π interactions (π-π stacked, π-alkyl, and alkyl) with key residues (Fig. 4B). Overall, demethoxycurcumin displayed strong interactions with key residues of Cdc2b, highlighting their potential as anti-amoebic agents.

**Table 2:** Binding affinity of curcumin and demethoxycurcumin against phosphatidylinositol-4-phosphate 3-kinase (Vps34) and Cell division control protein 2b (Cdc2b) from *Acanthamoeba* spp.

Compound	PubChem CID	VPS34 (AlphaFold ID AF-L8GS90-F1)				CDC2B (AlphaFold ID AF-L8HG09-F1)			
		Binding energy (kcal/mol)	Inhibition constant	No. of H-bonds	Amino-acid residues	Binding energy (kcal/mol)	Inhibition constant	No. of H-bonds	Amino-acid residues
Curcumin	969516	−6.26	25.75 μM	6	Two with VAL17, two with VAL97, ASN99, CYS100	−9.00	255.77 nM	5	LYS40, PHE91, LEU92, ASP95, ASP154
Demethoxycurcumin	5469424	−6.91	8.6 μM	1	LEU413	−8.07	1.21 μM	2	LEU92, ASP95
Control inhibitors									
Vemurafenib	42611257	−8.09	1.17 μM	4	LYS448, GLN1030, and two with CYS1053	−9.22	174.38 nM	2	Two with LEU92
MBQ-167	129896932	−8.67	443.48 nM	1	VAL1066	−9.24	169.97 nM	3	LEU92, two with ASP154
GSK-F1	57406702	−7.91	1.60 μM	6	ARG517, LYS987, ARG989, two with ASN1013, GLU1064	−8.83	339.34 nM	5	ASP93, GLN94, ASP95, THR98, ALA153
AG-1478	2051	−7.49	3.21 μM	3	MET360, SER402, LEU413	−7.40	3.75 μM	3	HIS170, two with VAL173

### 3.6 Molecular dynamics simulation analysis of demethoxycurcumin against Cdc2b proteins

**Fig. 5:**
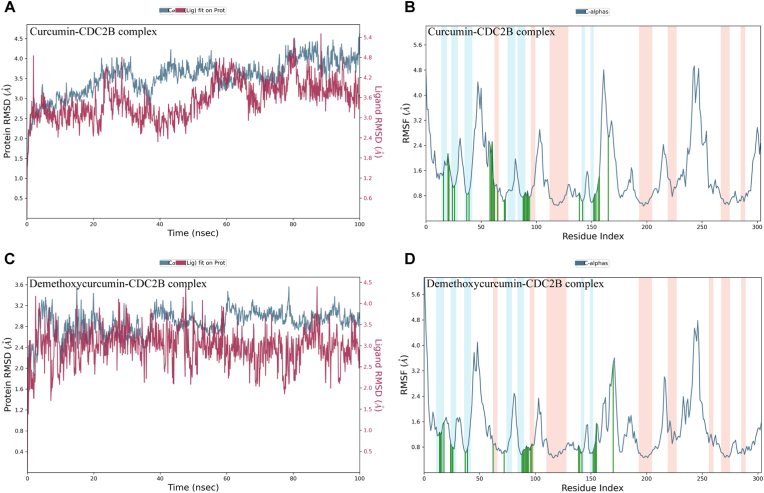
Molecular dynamics of Cdc2b in complex with curcumin and demethoxycurcumin. **A** Time evolution of backbone root mean square deviation (RMSD) of Cdc2b (*left axis*) and ligand RMSD relative to the protein (*right axis*) for the curcumin-Cdc2b complex over a 100-ns molecular dynamics simulation. **B** Per-residue root mean square fluctuation (RMSF) of Cdc2b in the curcumin-Cdc2b complex, highlighting regions of increased flexibility. Secondary structure elements are annotated, with α-helices, β-strands, and loop regions indicated. **C** Backbone and ligand RMSD profiles for the demethoxycurcumin-Cdc2b complex over 100 ns. **D** RMSF analysis of Cdc2b in the demethoxycurcumin-Cdc2b complex.

Curcumin and demethoxycurcumin emerged as promising Cdc2b inhibitors in docking studies, with demethoxycurcumin further evaluated through 100-ns molecular dynamics simulations alongside curcumin. RMSD analysis indicated that the curcumin-Cdc2b complex maintained stable protein dynamics (2.0–2.5 Å) during 40–100 ns with moderate ligand fluctuations (1.5–3.5 Å), whereas the demethoxycurcumin-Cdc2b complex showed slightly lower protein RMSD (1.8–3.2 Å) but broader ligand variation (1.6–3.6 Å), reflecting greater mobility (Fig. 5A and C). RMSF profiles revealed overall low residue fluctuations (< 2.0 Å), though demethoxycurcumin exhibited slightly higher peaks in flexible regions, while structured regions remained stable in both complexes (Fig. 5B and D).

**Fig. 6:**
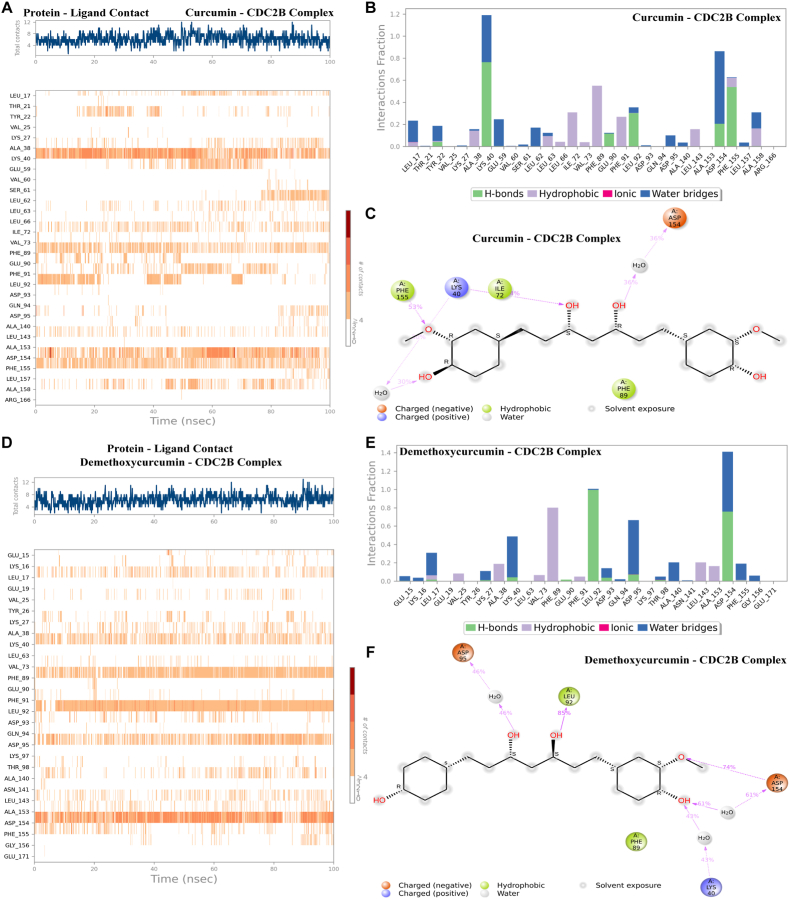
Protein-ligand interaction dynamics of *A*. *triangularis* Cdc2b complexes with curcumin and demethoxycurcumin. **A** Time-resolved protein-ligand contact analysis for the curcumin-Cdc2b complex over a 100-ns molecular dynamics simulation. The upper panel shows the total number of contacts over time, while the lower panel depicts residue-specific contact persistence throughout the simulation. **B** Interaction fraction analysis for curcumin bound to Cdc2b, showing the relative contributions of hydrogen bonds, hydrophobic interactions, ionic interactions, and water-mediated bridges across individual residues. **C** Two-dimensional interaction map of the curcumin-Cdc2b complex, highlighting key residue contacts, hydrogen bonds, water bridges, and solvent-exposed regions contributing to ligand stabilization. **D** Time-dependent protein-ligand contact profile for the demethoxycurcumin-Cdc2b complex, illustrating overall contact stability and residue-level interaction patterns during the 100-ns simulation. **E** Interaction fraction per residue for the demethoxycurcumin-Cdc2b complex, summarizing the frequency and type of intermolecular interactions maintained during the simulation. **F** Two-dimensional interaction diagram of demethoxycurcumin bound to Cdc2b, showing key stabilizing interactions, including hydrogen bonding, hydrophobic contacts, and water-mediated interactions.

The contact timeline analysis demonstrated that both curcumin and demethoxycurcumin maintained stable interactions with Cdc2b during the 100-ns simulation. Curcumin showed a consistent number of contacts, particularly with residues LYS40, PHE89, ASP15, and PHE155 (Fig. 6A), while demethoxycurcumin exhibited sustained but slightly more variable contacts involving LYS40, PHE89, LEU92, ASP95, and ASP154 (Fig. 6D). Overall, both ligands displayed persistent binding, with curcumin showing marginally greater stability. Interaction histograms (Fig. 6B and E) revealed diverse intermolecular contacts at the Cdc2b active site, including hydrogen bonds, hydrophobic interactions, ionic contacts, and water bridges. In the curcumin-Cdc2b complex (Fig. 6B and C), hydrogen bonds were observed at LYS40, GLU90, LEU92, ALA153, and PHE155, alongside hydrophobic interactions with ILE72 and PHE89. The demethoxycurcumin-Cdc2b complex (Fig. 6E and F) formed hydrogen bonds at LEU92 and ASP154, with hydrophobic contacts involving ALA38, PHE89, LEU143, and ALA153. These molecular dynamics findings are consistent with docking predictions, supporting the stable and specific binding of demethoxycurcumin at the Cdc2b active site.

### 3.7 Network pharmacology study of demethoxycurcumin against *Acanthamoeba* infection

**Fig. 7:**
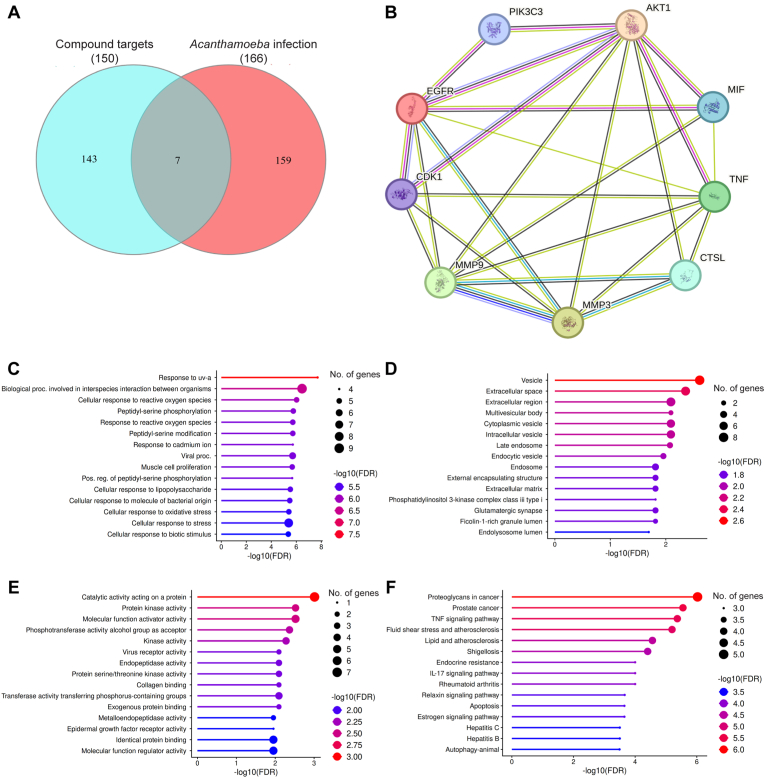
Network pharmacology and functional enrichment analysis of compound targets related to *A. triangularis* infection. **A** Venn diagram showing the overlap between predicted compound targets and genes associated with *A*. *triangularis* infection, identifying a subset of shared targets for downstream analysis. **B** Protein-protein interaction (PPI) network of the overlapping targets. Nodes represent proteins and edges denote known or predicted interactions, highlighting key hub proteins potentially involved in host-pathogen interactions and disease-related signaling. **C** Gene Ontology (GO) Biological Process enrichment analysis of the overlapping targets, with terms ranked by -log_10_(FDR). Color intensity ranges from blue to red, with red indicating more statistically significant enrichment. Dot size represents the number of genes associated with each term. **D** GO Cellular Component enrichment analysis, showing the predominant subcellular localizations of the overlapping targets. **E** GO Molecular Function enrichment analysis, summarizing the principal functional activities of the overlapping targets. **F** KEGG pathway enrichment analysis, identifying significantly enriched signaling and disease-related pathways associated with the overlapping targets.

To investigate how the predicted targets of demethoxycurcumin and curcumin interact during *Acanthamoeba* infection in humans, a protein-protein interaction (PPI) network was constructed using the STRING database. This network incorporated seven candidate proteins along with Vps34 (PIK3C3) and Cdc2b (CDK1). As shown in Fig. 7A, the Venn diagram illustrates the overlap between compound-associated targets (*n* = 150) and infection-related proteins (*n* = 166), identifying seven common targets. The PPI network, where nodes represent proteins, edges denote interactions, and line thickness reflects interaction strength, is shown in Fig. 7B. The identification of overlapping targets highlights molecular intersections through which the compounds may modulate host-pathogen interactions, providing potential therapeutic relevance and guiding the selection of candidate targets for further validation in anti-*Acanthamoeba* strategies. The hub genes identified in the PPI network included AKT1 (AKT Serine/Threonine Kinase 1, Protein Kinase B alpha), MIF (Macrophage Migration Inhibitory Factor), TNF (Tumor Necrosis Factor), CTSL (Cathepsin L), MMP3 (Matrix Metallopeptidase 3), MMP9 (Matrix Metallopeptidase 9), CDK1 (Cyclin-Dependent Kinase 1, also known as CDC2), EGFR (Epidermal Growth Factor Receptor), and PIK3C3 (Phosphatidylinositol 3-Kinase Catalytic Subunit Type 3, Vps34). These proteins are functionally diverse yet interconnected, participating in pathways related to inflammation, cell survival, autophagy, extracellular matrix remodeling, and immune regulation. For example, AKT1 and PIK3C3 are central to PI3K/AKT signaling and autophagy, TNF and MIF are key mediators of cytokine-driven immune responses, while MMP3, MMP9, and CTSL contribute to tissue invasion and remodeling relevant to *Acanthamoeba* pathogenesis. Collectively, the network suggests that demethoxycurcumin and curcumin may act through multi-target modulation, offering systems-level insights into their potential efficacy against *Acanthamoeba* infection.

GO biological process enrichment analysis indicated that the predicted targets of demethoxycurcumin and curcumin during *Acanthamoeba* infection are closely linked to stress responses, immune regulation, and phosphorylation-driven signaling. Key enriched terms included response to UV-A, cellular response to reactive oxygen species, and response to cadmium ion, emphasizing the compounds’ potential roles in oxidative stress modulation and detoxification (Fig. 7C). Complementary GO cellular component and molecular function analyses reinforced these findings. Enriched components such as vesicles, extracellular regions, multivesicular bodies, endosomes, and the endolysosome lumen suggest involvement in trafficking, secretion, and degradation processes (Fig. 7D). Molecular function enrichment highlighted catalytic activity on proteins, protein kinase activity, serine/threonine kinase activity, and phosphotransferase activity, underscoring the centrality of phosphorylation-dependent signaling (Fig. 7E). Additionally, enrichment in virus receptor activity, collagen binding, metalloendopeptidase activity, and epidermal growth factor receptor activity points to functions in pathogen recognition, extracellular matrix remodeling, and receptor-mediated signaling. Taken together, these results suggest that demethoxycurcumin and curcumin exert broad, multi-target effects by modulating oxidative stress responses, immune pathways, and kinase-mediated signaling, which may underlie their inhibitory activity against *Acanthamoeba* infection. KEGG enrichment analysis of the *Acanthamoeba* dataset revealed strong associations with diverse signaling and disease-related pathways. Notably, the enrichment of autophagy alongside apoptosis, TNF signaling, and infection-related pathways indicates that *Acanthamoeba* may rely on conserved stress-response and survival mechanisms to withstand hostile conditions (Fig. 7F). Autophagy, in particular, appears to facilitate cellular remodeling and persistence under nutrient deprivation or immune challenge, consistent with its established role in protozoan pathogenicity. These findings position autophagy as a key regulatory pathway in *Acanthamoeba*, intersecting with host-pathogen interactions and providing a mechanistic foundation for potential therapeutic interventions against amoebic infections.

## 4 Discussion

Antimicrobial activity of the major curcumin derivatives, i.e. curcumin (Nisar et al., 2015), demethoxycurcumin (Noor et al., 2020), bisdemethoxycurcumin, and curcuminoids (Afrida et al., 2021), has been reported. Curcumin has been shown to have anti-*Acanthamoeba* action against the well-known T4 genotype member *Acanthamoeba castellanii* by numerous research groups (El-Sayed et al., 2012; Saeed et al., 2022). Our recent study has revealed anti-*Acanthamoeba* activity of curcumin against *A*. *triangularis* WU19001, another member of the T4 (Boonhok et al., 2022). In this study, demethoxycurcumin, bisdemethoxycurcumin, and curcuminoids were tested for their amoebic activity. Among these compounds, demethoxycurcumin exhibited the highest amoebicidal activity against *A*. *triangularis*, whereas bisdemethoxycurcumin and curcuminoids had lower amoebicidal activity. Antagonistic effects in curcuminoids might be a cause of low amoebicidal activity. Demethoxycurcumin was also evaluated against the pathogenic strain *A. castellanii* ATCC 50739, and its MIC was higher than that observed for *A. triangularis*. This finding may suggest that the pathogenic strain exhibits greater adaptability or tolerance under drug pressure compared with non-pathogenic strains or natural isolates of *Acanthamoeba*. This common trait of microbes can be found in a variety of microorganisms, such as bacteria, parasites, including amoeba (Bansal et al., 2006). However, the underlying mechanism for drug resistance requires further investigation. In this study, the mechanism of demethoxycurcumin-induced *Acanthamoeba* cell death was not characterized. Nevertheless, in other studies, demethoxycurcumin was noted to induce apoptosis in many cell types (Ko et al., 2015). Aqeel et al. (2012) discovered that demethoxycurcumin did not influence the level of proteases during *A*. *castellanii* treatment, despite the fact that the mechanism of action for the anti-*Acanthamoeba* feature of demethoxycurcumin had not yet been defined. Moreover, the demethoxycurcumin was documented as a potent inhibitor of P-Type ATPases. These are the integral membrane proteins capable of using the energy-rich ATP molecule to actively pump ions and small organic molecules across biological membranes found in both bacteria and eukaryotic cells (Bublitz et al., 2011). The similar potent mechanisms may also explain the effect of demethoxycurcumin in killing *A*. *triangularis*. However, further investigation aimed at reducing the effective concentration to the micromolar or nanomolar range, potentially through side-chain modification or structural optimization, will be important before advancing demethoxycurcumin toward therapeutic applications. Several complementary methods were employed to assess *Acanthamoeba* viability, including colorimetric and fluorometric assays, i.e. MTT, resazurin sodium salt, and PrestoBlue®, as well as the classical trypan blue exclusion assay (Heredero-Bermejo et al., 2013). When testing standard drug control, chlorhexidine, and some natural products, resazurin sodium salt and PrestoBlue® exhibited high sensitivity, while MTT generated lower signal intensity but remained sufficient to determine IC_50_ values. In contrast, treatment with demethoxycurcumin resulted in uniformly low signals across all three metabolic assays, preventing reliable discrimination of optical density differences among treatment conditions and precluding accurate IC_50_ determination. This limitation is likely due to the intrinsic color and redox properties of curcumin derivatives, which may interfere with assay chemistry or optical signal detection (Bruggisser et al., 2002; Tapia-Rojas et al., 2024). Consequently, we reverted to the trypan blue exclusion assay, a dye-based method independent of metabolic activity and colorimetric interference. Importantly, viability data obtained using trypan blue were consistent with trends observed in the metabolic assays for other compounds, supporting its reliability for assessing *Acanthamoeba* viability in the context of curcumin derivative treatment.

To further elucidate the ultrastructural effects of curcumin derivatives on *Acanthamoeba*, scanning electron microscopy (SEM) was employed to examine surface morphology following treatment. In the untreated controls, both trophozoites and cysts clearly displayed the characteristic external features of *Acanthamoeba* (Jongwutiwes et al., 2000; Garajová et al., 2019; Sangkanu et al., 2021). Untreated trophozoites exhibited an irregular, pleomorphic surface with numerous acanthopodia, appearing as spine-like projections extending from the cell surface, while the plasma membrane between projections remained continuous and intact. In contrast, untreated cysts of *A*. *triangularis* predominantly showed a triangular or polygonal external shape with a rigid cyst wall, distinct surface ridges and venation patterns, and, in some cysts, a pore-like opening (ostiole) on the cyst surface. Although we observed in our laboratory that cyst shape can vary depending on the culture medium and encystation-inducing conditions, both trophozoites and cysts consistently retained their key SEM-identifiable characteristics, in agreement with previous ultrastructural reports. Following treatment, marked surface alterations were observed. In a previous study by Mitsuwan et al. (2020b), SEM analysis of *Acanthamoeba* treated with *C*. *longa* extract or curcumin revealed pronounced morphological changes in trophozoites, including loss of acanthopodia, pore formation on the parasite membrane, and a transition toward a spherical shape, indicating membrane damage. Consistent with these findings, our investigation demonstrated that curcumin derivatives similarly induced membrane pore formation, acanthopodia loss, and rounding of trophozoites. In cysts, treatment with curcumin derivatives resulted in cell swelling, surface deformation, and loss or blurring of cyst wall venation, suggesting disruption of external cyst wall architecture. Several of these surface features were comparable to those reported following treatment with other plant extracts, such as *Cassia angustifolia* (Boonhok et al., 2021). Collectively, these SEM observations demonstrate that curcumin derivatives exert their anti-*Acanthamoeba* effects through direct disruption of trophozoite surface structures and cyst wall integrity.

*Acanthamoeba* encystation refers to a transformation mechanism of *Acanthamoeba* trophozoite to cyst under stress conditions (Siddiqui and Khan, 2012). Several intracellular pathways have been implicated in *Acanthamoeba* encystation, including those involved in glycolysis, proteolysis, and actin dynamics (Bouyer et al., 2009). Specific parasite proteins reported to be involved include cysteine proteases (Leitsch et al., 2010; Moon et al., 2012), serine protease (Dudley et al., 2008; Moon et al., 2008), Shwachman-Bodian-Diamond syndrome protein (Wang et al., 2021), and autophagy-related proteins (Atg) (Moon et al., 2009; Kim et al., 2015). However, the coordination of these pathways or proteins under different stress conditions is poorly understood. Autophagy is an intracellular degradative pathway and known as a stress-sensing mechanism, responding quickly to stress signals, e.g. starvation, rapamycin, cytokines, reactive oxygen species, etc. (Yorimitsu and Klionsky, 2005). In yeasts and mammals, more than 30 autophagy-related proteins have been discovered, and these proteins are mainly required for an autophagosome formation and some play additional roles in autophagosome maturation as well as degradation of autophagic contents (Yorimitsu and Klionsky, 2005). However, in *Acanthamoeba*, a small number of Atg proteins were identified, i.e. Atg3, Atg8b, Atg12, and Atg16 and reported to be associated with *Acanthamoeba* encystation. Starvation or a nutrient-depleted condition is a well-known condition to induce autophagy in several models for autophagy study (Mejlvang et al., 2018). Starvation of *Acanthamoeba* trophozoite culture was able to induce *Acanthamoeba* encystation. However, the degree of the encystation depends on the medium formulation, time of treatment, as well as *Acanthamoeba* species (Sohn et al., 2017). Encystation efficiency is a key parameter in *Acanthamoeba* studies, and fully mature cysts are typically formed after 5–7 days under encystation-inducing conditions (Coulon et al., 2010). In the present study, however, our primary aim was to evaluate the amoebicidal activity of pure compounds at an early time-point (24 h); for consistency across assays, encystation was therefore assessed at 24 h. Under these conditions, encystation was clearly initiated, with *c*.50% of cells adopting cyst morphology, and cysts with rigid cyst walls were readily observed by light microscopy, indicating progression toward cyst maturation. We also compared Neff’s encystment medium and PAS medium and found no clear difference in cyst formation or morphology at 24 h. While Neff’s encystment medium is more effective for generating fully mature cysts over prolonged incubation (5–7 days), both media were sufficient to induce early encystation within 24 h (Boonhok et al., 2021). Thus, the starvation by PAS medium in this study is appropriate for assessing early encystment dynamics and compound-mediated inhibition while maintaining consistency with the amoebicidal assays. Our previous study found that under starvation with 3 MA, a phosphoinositide 3-kinase (PI3K) inhibitor that inhibits Vps34 activity (Wu et al., 2010), the percentage of cysts under this condition was significantly reduced by *c*.20%. In the presence of curcumin under starvation, the percentage of cysts was very low, *c*.1–2% (Boonhok et al., 2022). However, in the presence of curcumin with 3 MA, cyst formation was not completely inhibited as expected. Thus, we hypothesized that curcumin and 3 MA may target the Vps34 and lead to *Acanthamoeba* trophozoite arrest in the surviving amoebas. Together with the results in this study found that all tested curcumin derivatives demonstrated the percentage of cysts of less than 3% upon treatment with sublethal doses, and demethoxycurcumin was the most potent in inhibiting the cyst formation or trophozoite arrest induction. Altogether, the data indicate similar biological activities of curcumin derivatives, and the *Acanthamoeba* Vps34 becomes a target molecule in our study.

At the sublethal dose of curcumin derivatives treatment, the molecular analysis by real-time PCR was carried out to assess the autophagy-related gene expression, i.e. ATG3, ATG8b, ATG12, and ATG16 in the surviving parasites in light of the inhibition of cyst formation by demethoxycurcumin as well as the association between starvation and *Acanthamoeba* autophagy. As expected, in the presence of demethoxycurcumin, the expression of *Acanthamoeba* ATG3, ATG8b, ATG12, and ATG16 mRNA was not upregulated and maintained at the basal level. The results may indicate the inhibitory effect of demethoxycurcumin on *Acanthamoeba* autophagy. However, the precise mechanism remains unclear and warrants further investigation to determine whether demethoxycurcumin inhibits autophagy, leading to cyst suppression or trophozoite arrest, or whether these effects occur through an alternative pathway. Curcumin at low concentrations has been revealed to modulate autophagy, either through induction or inhibition (Shakeri et al., 2019). Different outcomes are varied depending on the models studied, including doses of curcumin and the type of cells. The effect of demethoxycurcumin on autophagy induction was also mentioned in one of several studies in human lung cancer cells (Chen and Wu, 2021). However, the effect of this compound on autophagy inhibition has never been reported.

The molecular analysis of other *Acanthamoeba* encystation-related genes, such as aminopeptidase and Shwachman-Bodian-Diamond syndrome, was also performed in the present study. The expression of aminopeptidase was also at the basal level, whereas the SBDS mRNA was slightly higher at the beginning of post-treatment; however, the expression of this gene later declined to the basal level. This may indicate other activities of this protein under normal conditions or indirectly indicates the inhibitory effect of demethoxycurcumin on the SBDS protein activity. Additionally, the cell cycle is another pathway involved in cell proliferation. In *Acanthamoeba*, the cell division control gene 2b (Cdc2b) was recently identified (Mengue et al., 2016). Under demethoxycurcumin treatment, the Cdc2b mRNA expression was similar to that of SBDS in which the expression was very low at 24 h post-treatment. Curcumin treatment resulted in cell cycle arrest at G1/S and G2/M phases in human osteosarcoma (HOS cells) (Lee et al., 2009), and another molecular analysis reported the downregulation of several cell cycle proteins, for example, cell cycle-dependent kinase 2 (CDK2), cyclins, and transcription factor E2F1 upon curcumin treatment (Li et al., 2022). We thus hypothesized that Cdc2b protein could be another target of demethoxycurcumin to demonstrate trophozoite arrest in *Acanthamoeba*.

Studies on demethoxycurcumin are not in-depth and broad compared to curcumin in terms of its biological activities related to human health and diseases. Curcumin contains hydrogen bond-forming functional groups, hydrophobic aromatic rings, and a flexible linker region, which together influence its solubility and enable interactions with various biomolecules through non-covalent and also covalent binding (Sala de Oyanguren et al., 2020). The sole way that demethoxycurcumin is different from regular curcumin is by the number of methoxy groups on the aromatic rings. However, the binding pockets of these compounds on the same protein might be different. Curcumin and curcumin derivatives can modulate many lipids and membrane proteins as well as directly interact with several proteins in the cytoplasm (Duda et al., 2020). Another finding showed that curcumin targeted the endoplasmic reticulum (ER), causing the ER to expand, in Huh-7 cells, which is another piece of evidence on the curcumin effect and autophagy (Sala de Oyanguren et al., 2020). Consistent with previous reports, our findings highlight the dual activity of demethoxycurcumin, demonstrating both amoebicidal effects and growth arrest of surviving *Acanthamoeba* trophozoites. The observed disruption of membrane integrity following exposure to demethoxycurcumin or curcumin further suggests that these compounds may penetrate trophozoites, interact with intracellular targets, and subsequently induce trophozoite arrest. We thus hypothesized that curcumin and/or demethoxycurcumin may target *Acanthamoeba* autophagy or cell cycle pathways to obtain the outcomes above. We then selected two candidates for *Acanthamoeba* proteins, i.e. Vps34 and Cdc2b to analyze the possible interaction with curcumin and demethoxycurcumin by molecular docking and molecular dynamics analysis.

*In silico* molecular docking provides a rapid approach to predict interactions between bioactive compounds and candidate target proteins. In this study, both demethoxycurcumin and curcumin exhibited binding affinity toward two *Acanthamoeba* proteins involved in encystation-related processes: Vps34, a class III phosphoinositide 3-kinase regulating autophagy; and Cdc2b, a putative cyclin-dependent kinase associated with cell-cycle progression. The binding affinity of a compound is determined by the type and number of interactions formed within the protein binding pocket, including hydrogen bonds and other stabilizing contacts (Abd El-Aziz et al., 2022). For Vps34, demethoxycurcumin and curcumin formed multiple hydrogen bonds and interacted with partially distinct binding pockets, with demethoxycurcumin sharing some overlapping residues with the GSK-F1 inhibitor site. However, these interactions were less consistent compared with those observed for Cdc2b. Notably, Cdc2b showed more favorable and coherent docking results, with demethoxycurcumin displaying stronger binding affinity than curcumin and forming stable hydrogen-bond networks within a binding pocket that overlapped with known Cdc2b inhibitors. These findings suggest that Cdc2b may represent a more promising intracellular target of demethoxycurcumin. Although curcumin and demethoxycurcumin possess relatively hydrophobic structures, only a limited number of hydrophobic residues were identified within the predicted binding pockets, indicating that hydrophobic interactions may play a minor role in target engagement. Instead, polar interactions and hydrogen bonding, potentially mediated by the phenolic groups of demethoxycurcumin, appear to be the dominant contributors. Consistent with this notion, the phenolic moieties of curcumin have been reported to mediate antimicrobial activity through specific molecular interactions, such as binding to bacterial lipopolysaccharide (Hettiarachchi et al., 2022).

Based on these findings, Cdc2b was selected for further investigation using molecular dynamics simulations to evaluate the stability and persistence of the protein-ligand complex over time. *Acanthamoeba* encystation is a critical survival mechanism that allows the parasite to withstand adverse environmental conditions, and Cdc2b, a putative cyclin-dependent kinase, has been implicated as a key regulator of this process by controlling cell cycle progression and signaling pathways essential for cyst formation (Samba-Louaka, 2023). Inhibition of Cdc2b, therefore, represents a promising therapeutic strategy to disrupt encystation and reduce the persistence of *Acanthamoeba* infections. Molecular docking is a computational technique used to predict the optimal orientation (binding pose) and binding affinity of a ligand when interacting with a receptor or protein. This approach provides a rapid and efficient screening method for identifying promising lead compounds in drug discovery (Agu et al., 2023). The molecular docking and dynamics analyses collectively highlight demethoxycurcumin as a promising candidate for targeting *Acanthamoeba* encystation through inhibition of Cdc2b, despite showing slightly weaker binding affinity compared to curcumin. While curcumin demonstrated stronger binding energy and more extensive hydrogen bonding, interactions of demethoxycurcumin with critical residues such as LEU92 and ASP95, coupled with diverse stabilizing contacts including π interactions and hydrophobic forces, suggest functional relevance even with fewer hydrogen bonds. The high affinity of a drug or compounds depends on the type and amount of the interaction/bonding that occurs within the binding pocket of the protein (Abd El-Aziz et al., 2022). In previous studies, demethoxycurcumin significantly reduced *A*. *castellanii* adhesion (73%), inhibited host cell cytotoxicity (86%), and showed amoebicidal activity (25%) at 100 μg, highlighting its promising anti-amoebic potential (Aqeel et al., 2012). Molecular docking studies in previous work have reported that dimethylamino curcuminoid derivatives display enhanced anti-inflammatory activity compared to parent curcumin, with favorable interactions against COX-1 and COX-2 enzymes (Banuppriya et al., 2016). Previous studies employing computational approaches against *Acanthamoeba* infection revealed that venadaparib achieved the highest docking score and binding free energy (−89.28 kcal/mol), closely followed by AZ9482 (−92.13 kcal/mol), with both compounds stabilized through strong hydrogen bonding and aromatic stacking interactions within the *Acanthamoeba* PARP binding pocket (Chen et al., 2024). The molecular dynamics simulations further reinforced and confirmed our docking findings, showing that demethoxycurcumin maintained stable binding throughout the 100-ns trajectory, with slightly greater ligand mobility but consistent contacts at the Cdc2b active site with LEU92 and ASP154 (Fig. 6E). This dynamic behavior may reflect flexibility that allows adaptive binding, complementing the docking predictions. MD simulations are a crucial computational tool in modern drug discovery, providing atomic-level insights into the dynamic interactions between potential drugs (ligands) and their biological targets (proteins) (De Vivo et al., 2016). Additional proteins associated with autophagy or the cell cycle may also play a role and represent potential targets for curcumin or its derivatives, warranting investigation in future studies. Overall, while curcumin demonstrates slightly greater stability, the sustained interactions and favorable binding profile of demethoxycurcumin highlight its promise as an anti-amoebic agent, meriting further experimental validation. The network pharmacology analysis highlighted that demethoxycurcumin exerts its anti-acanthamoebic potential through multi-target modulation of host-pathogen interactions, with hub proteins such as AKT1, TNF, MIF, EGFR, CDK1, MMP3, MMP9, CTSL, and PIK3C3 (Fig. 7B) emerging as central regulators of inflammation, autophagy, immune signaling, and extracellular matrix remodeling. GO enrichment further supports the involvement of oxidative stress responses, phosphorylation-driven signaling, and vesicle-mediated trafficking, suggesting that demethoxycurcumin may attenuate amoebic pathogenicity by disrupting stress adaptation and immune evasion mechanisms. KEGG pathway analysis reinforced the importance of autophagy, apoptosis, and TNF signaling in *Acanthamoeba* survival, positioning autophagy as a critical regulatory axis that intersects with host defence pathways. Collectively, these findings provide systems-level evidence that demethoxycurcumin acts through diverse molecular intersections, underscoring its promise as a candidate for therapeutic intervention against *Acanthamoeba* infection and warranting further experimental validation.

## 5 Conclusions

Demethoxycurcumin, a bioactive curcumin derivative, demonstrated potent anti-*Acanthamoeba* activity, including amoebicidal effects and sustained trophozoite arrest under nutrient starvation. These effects were consistent with, but more pronounced than, those observed for other curcumin derivatives. Our findings indicate that demethoxycurcumin interferes with key regulatory processes governing *Acanthamoeba* survival, particularly autophagy and cell-cycle control. Computational analyses suggested that demethoxycurcumin can engage the autophagy-related kinase Vps34 and the cell-cycle regulator Cdc2b with binding affinities comparable to established inhibitors, providing a plausible molecular basis for its biological activity. Although further cell-based and functional assays are required to validate these interactions and delineate the downstream mechanisms of parasite death, this study identifies curcumin derivatives, particularly demethoxycurcumin, as promising multi-target modulators of *Acanthamoeba* growth and encystation. Collectively, these findings advance our understanding of *Acanthamoeba* biology and support the further development of curcumin-based compounds as potential therapeutic agents against *Acanthamoeba* infections.

## Ethical approval

Not applicable.

## CRediT authorship contribution statement

**Rachasak Boonhok:** Conceptualization, Methodology, Investigation, Data curation, Formal analysis, Visualization, Writing - original draft, Writing - review & editing. **Wilaiwan Senghoi:** Investigation, Data curation, Formal analysis, Visualization, Writing - review & editing, Funding acquisition. **Aman Tedasen:** Investigation, Formal analysis, Writing - review & editing. **Suthinee Sangkanu:** Investigation, Data curation, Writing - review & editing. **Chooi Ling Lim:** Methodology, Resources, Writing - review & editing. **Maria de Lourdes Pereira:** Conceptualization, Supervision, Writing - review & editing, Funding acquisition. **Mohammed Rahmatullah:** Writing - review & editing, Funding acquisition. **Polrat Wilairatana:** Methodology, Resources, Writing - review & editing. **Christophe Wiart:** Methodology, Resources, Writing - review & editing. **Karma G. Dolma:** Investigation, Writing - review & editing. **Alok K. Paul:** Investigation, Writing - review & editing. **Madhu Gupta:** Investigation, Writing - review & editing. **Md Atiar Rahman:** Resources, Writing - review & editing. **Kingkan Bunluepuech:** Resources, Writing - review & editing. **Shanmuga Sundara:** Methodology, Resources, Writing - review & editing. **Tooba Mahboob:** Methodology, Resources, Writing - review & editing. **Veeranoot Nissapatorn:** Conceptualization, Supervision, Writing - review & editing, Funding acquisition.

## Funding

This study was supported by The Royal Patronage of Her Royal Highness Princess Maha Chakri Sirindhorn-Botanical Garden of the Walailak University, Nakhon Si Thammarat, under the project entitled “Medicinal Thai native plants against *Acanthamoeba triangularis* and co-infection of *Acanthamoeba* with other microorganisms” (RSPG-WU-26/2566) (Thailand), awarded to Veeranoot Nissapatorn. Maria de Lourdes Pereira thanks the national funds (Portugal) through the FCT/MEC (PIDDAC), project CICECO-Aveiro Institute of Materials, UIDB/50011/2020, UIDP/50011/2020, and LA/P0006/2020. The funders had no role in study design, data collection and analysis, decision to publish, or preparation of the manuscript.

## Declaration of competing interests

The authors declare that they have no known competing financial interests or personal relationships that could have appeared to influence the work reported in this paper.

## Data Availability

The data supporting the conclusions of this article are included within the article and its supplementary file.
