# Supplementary material for: Demethoxycurcumin exhibits amoebicidal activity against Acanthamoeba triangularis trophozoites and cysts and inhibits encystation
Source: Curr Res Parasitol Vector Borne Dis. 2026 Feb 16;9:100362. doi: 10.1016/j.crpvbd.2026.100362 (PMC13080493; doi:10.1016/j.crpvbd.2026.100362)
Supplement: Multimedia component 1 [file mmc1.pdf]

Supplementary materials for*:*

Demethoxycurcumin exhibits amoebicidal activity against *Acanthamoeba triangularis* trophozoites and cysts and inhibits encystation

Rachasak Boonhok[a,*], Wilaiwan Senghoi[a], Aman Tedasen[a], Suthinee Sangkanu[b], Chooi Ling Lim[c], Maria de Lourdes Pereira[d], Mohammed Rahmatullah[e], Polrat Wilairatana[f], Christophe Wiart[g], Karma G. Dolma[h], Alok K. Paul[i], Madhu Gupta[j], Md. Atiar Rahman[k], Kingkan Bunluepuech[a], Shanmuga Sundara[l], Tooba Mahboob[m], Veeranoot Nissapatorn[n]

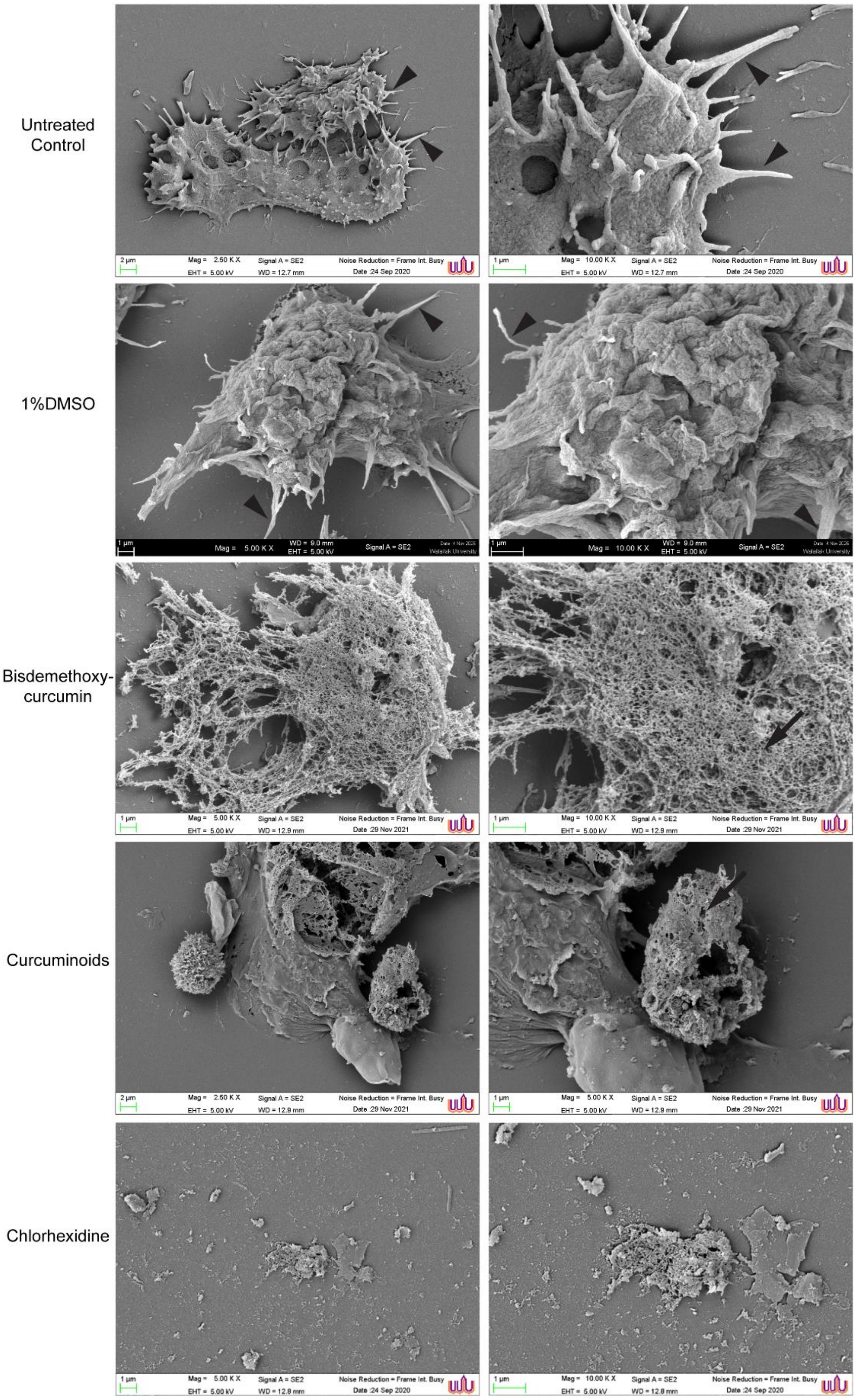

Supplementary Figure S1. SEM imaging of *A. triangularis* trophozoites treated with curcumin derivatives. *Acanthamoeba* trophozoites were treated with 1000 µg/ml of the compounds for 24 h. The amoebas were then processed for SEM analysis. Arrowheads indicate acanthopodia whereas arrows indicate porous membrane.

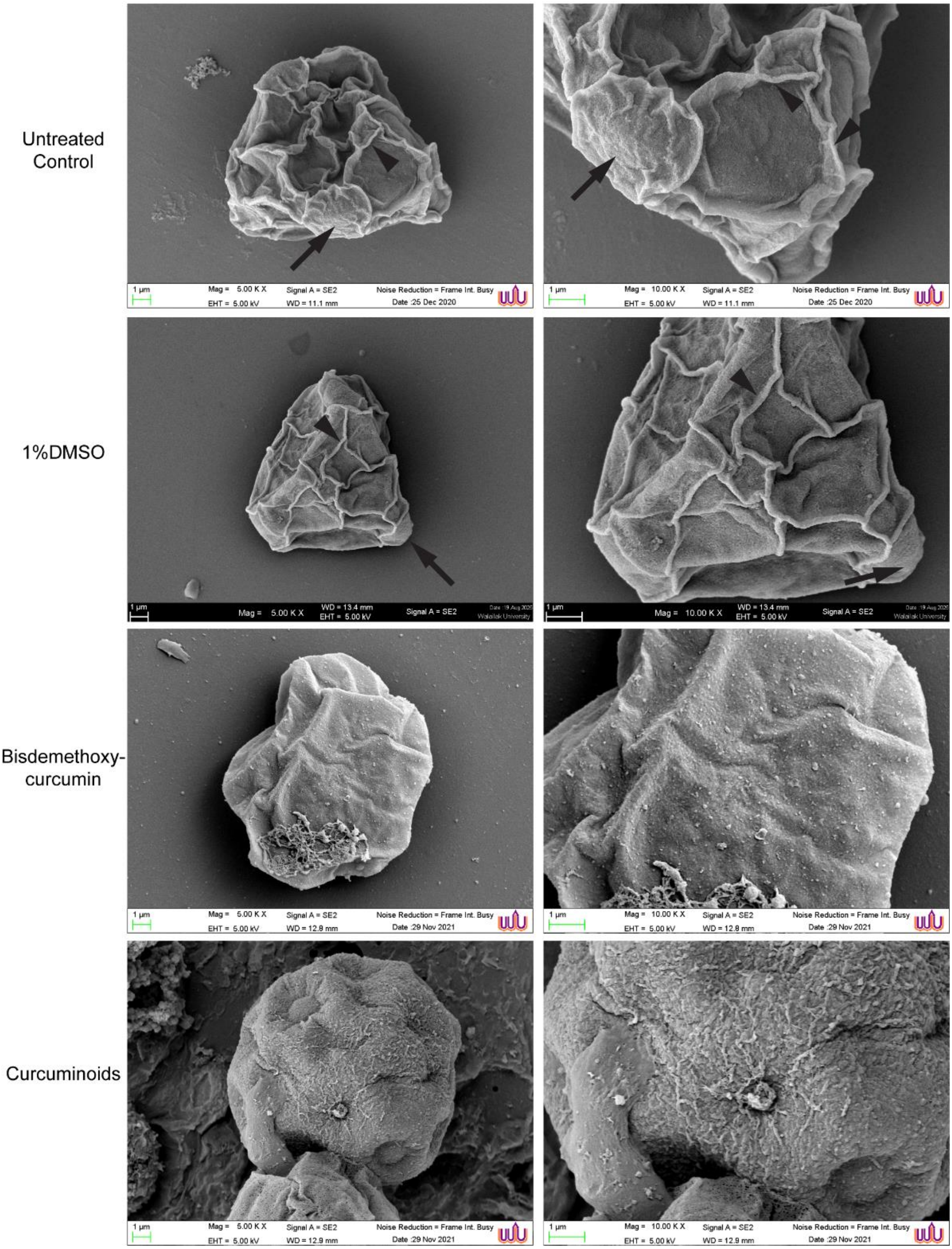

Supplementary Figure S2. SEM imaging of *A. triangularis* cysts treated with curcumin derivatives. *Acanthamoeba* cysts were treated with 1000 µg/ml of the compounds for 24 h. The amoebas were then processed for SEM analysis. Arrows indicate ostiole with circular pronounced edges, whereas arrowheads indicate venation.

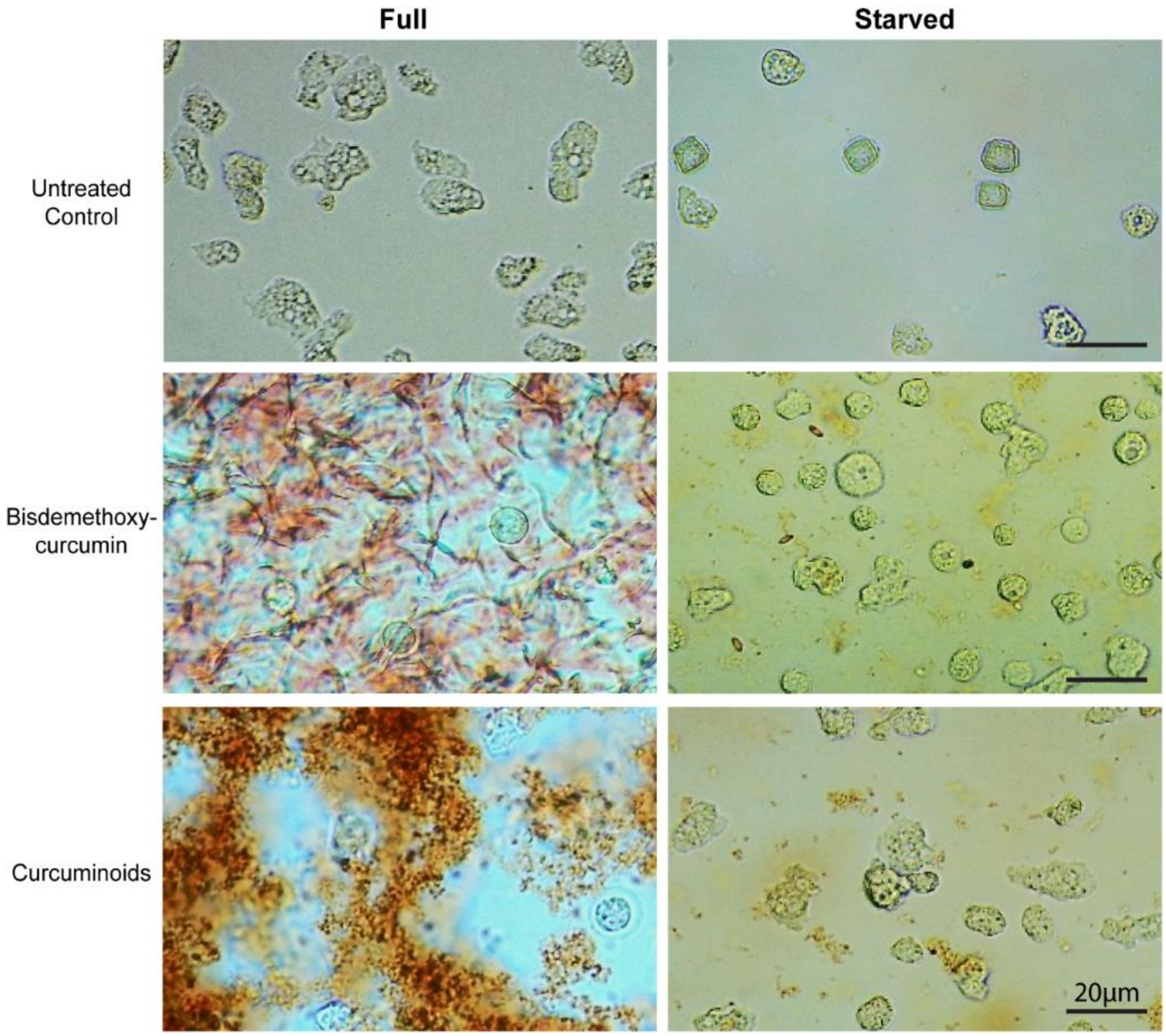

Supplementary Figure S3. ***A. triangularis*** **cyst formation under nutrient-rich and -depleted conditions in the presence of curcumin derivatives.** *Acanthamoeba* trophozoites were treated with curcumin derivatives at sublethal doses for 24 h under full (left) or starved (right) condition. The images were then collected by inverted microscope. Scale-bars: 20 µm.

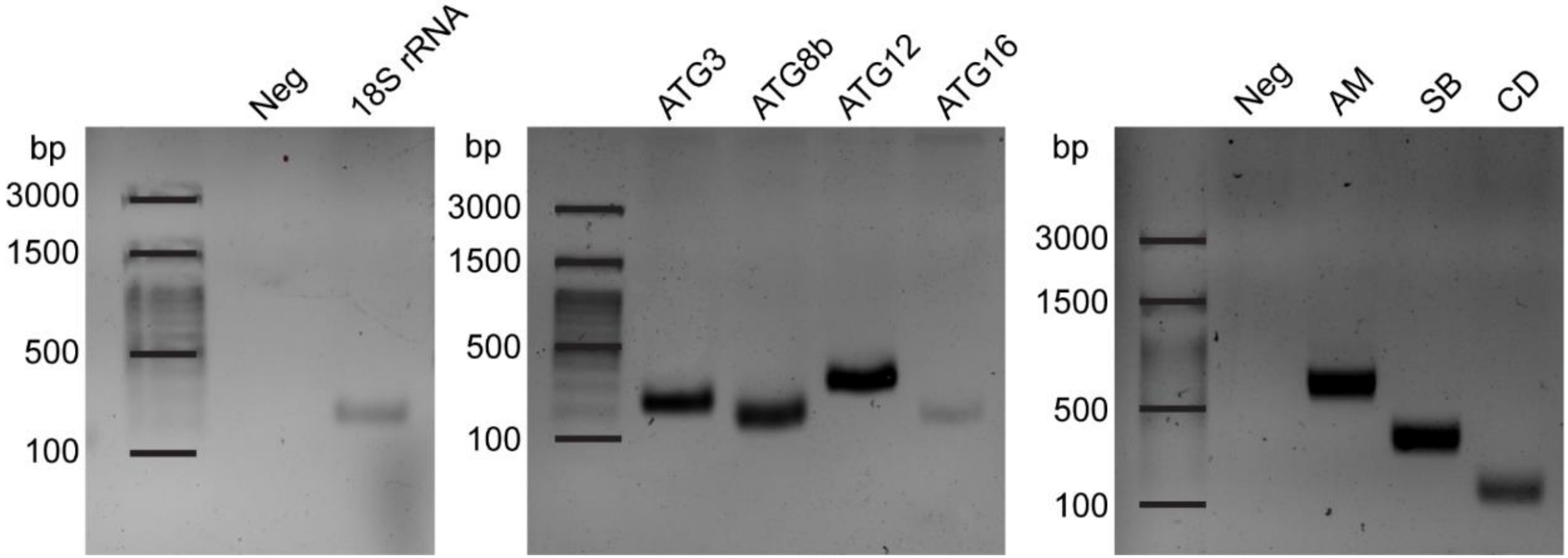

Supplementary Figure S4. Validation of PCR primers by conventional PCR and gel electrophoresis. PCR primers were confirmed their specificity by conventional PCR and *A. triangularis* DNA was used as DNA template. All PCR products were run on 1.5% agarose gel. DNA ladder (DM2300, SMOBIO®) was included to indicate the product size in bp. *Abbreviations*: Neg, negative control; ATG, autophagy-related gene; AM, aminopeptidase; SB, Shwachman-Bodian-Diamond syndrome; CD, Cell division control protein 2b.

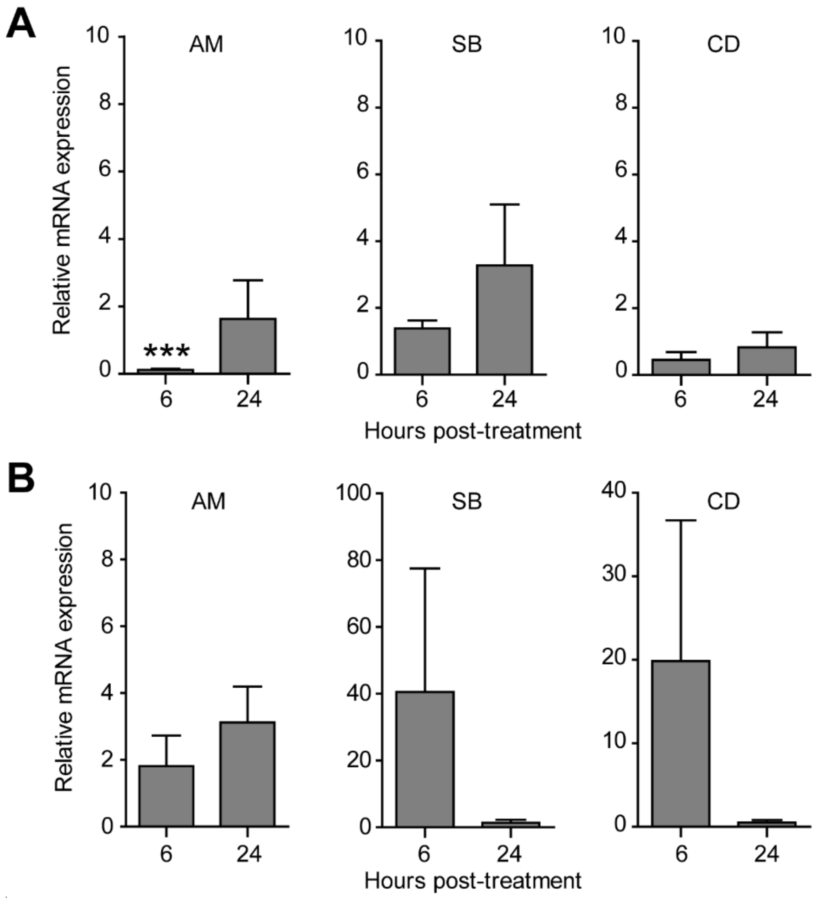

Supplementary Figure S5. Transcriptional expression of other encystation-related genes and cell division control gene. The cDNA samples were shared with ATG mRNA analysis. The qPCR was performed and 18S rRNA was used as a reference gene. Encystation-related genes (non-ATG genes) i.e. aminopeptidase (AM) and Shwachman-Bodian-Diamond syndrome (SB) as well as cell division control gene 2b (Cdc2b, CD) were analyzed. **A** Expression level of the mRNAs under full condition + demethoxycurcumin. **B** Expression level of the mRNAs under nutrient starvation + demethoxycurcumin. Data were obtained from 3 independent experiments. Bar graphs represent mean ± SEM. ***$P < 0.001$.

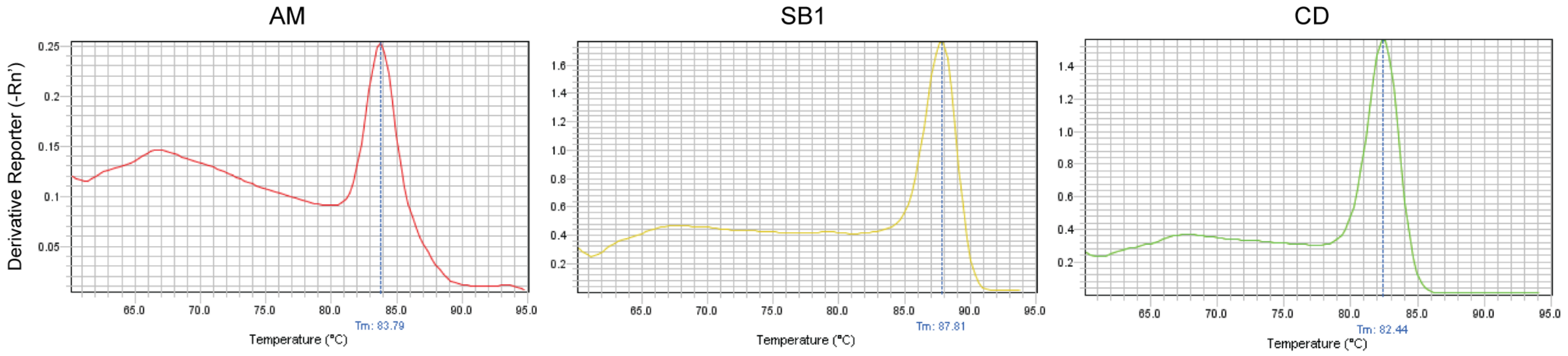

Supplementary Figure S6. Representative melting curve analysis of qPCR amplicons. Dissociation (melting) curves of representative qPCR products for AM, SB1, and CD are shown. The AM and SB1 amplicons exceed 250 bp, whereas CD represents a shorter target.

Supplementary Table S1. Determination of minimal inhibitory concentration of demethoxycurcumin against *A. castellanii* ATCC50739 trophozoites at 24 hours.

**Demethoxycurcumin (µM)**	**%*A. castellanii* survival**	**Chlorhexidine (µM)**	**%*A. castellanii* survival**
3026.45*	4.41 ± 0.00	126.62	0.00 ± 0.00
1513.23	20.96 ± 1.56	63.31*	2.21 ± 1.04
756.61	38.24 ± 1.04	31.66	23.90 ± 3.64
378.31	52.21 ± 3.12	15.83	21.32 ± 0.00
189.15	62.13 ± 13.00	7.91	22.79 ± 0.00
94.58	87.13 ± 3.64	-	-

*MIC of demethoxycurcumin and chlorhexidine against *Acanthamoeba castellanii* ATCC50739 trophozoites were 3026.45 µM and 63.31 µM, respectively.

Supplementary Table S2. List of primers for quantitative PCR.

Gene	GenBank accession No.	Forward (F)	Reverse (R)	*Acanthamoeba* spp.	Reference
**ATG3**	GU270859	5’-GCGCACGTACGATATCTCCATC-3’	5’-ATGAACACTTGGTTCGGCGTC-3’	*A. castellanii*	Moon et al., 2011
**ATG8b**	KC524507.1	5’-CCGAGTTCCTGTGATCGTTGA-3’	5’-AGCTGTGTGACGGCAATATCG-3’	*A. castellanii*	Moon et al., 2013
**ATG12**	HQ830265.1	5’-CCAGTCGAAGAGTACATGAAAGA-3’	5’-GCGAAGGAAGTCCACGA-3’	*A. castellanii*	Kim et al., 2015
**ATG16**	FJ906697	5’-AGCTTGACTTCCATCACGCTGA-3’	5’-TGTTTGAGGTTGGCCCGAA-3’	*A. castellanii*	Song et al., 2012
**Aminopeptidase**	ACA1_264610	5′-TGGATCGAGTTCAAGGAGGG-3′	5′-GCCAGGTGTGCTCGAAGAAGT-3′	*A. castellanii*	Huang et al., 2017
**Shwachman-Bodian-Diamond syndrome**	ACA1_142090	5′-CGAAGGACTGAAGGTGAGGT-3′	5′-GGACAGGATCTTTCGGATCA-3′	*A. castellanii*	Wang et al., 2021
**Cell division control protein 2b**	XM_004353658.1	5′-ATGCAAGCCAAACCCAGTC-3'	5′-GAATCGCTGGTTCTCGGTATC-3′	*A. castellanii*	Mengue et al., 2016
**18S rRNA**	-	5′-TCCAATTTTCTGCCACCGAA-3′	5′-ATCATTACCCTAGTCCTCGCGC-3′	*A. castellanii*	Song et al., 2012; Moon et al., 2008

Supplementary Table S3. *Acanthamoeba triangularis* DNA sequence by Sanger sequencing.

**Target gene** (accession No.)	**Primer**	**DNA sequence**	**Product length (bp)**	**% Identity**[a]
**Aminopeptidase** ACA1_264610	F	5’-GCGGGTGCTCGAGATGCAGATCCAGCAGCTCATCAACGAGAAGGCGCACTTCATGG ACGTCACTGACCACCCCGACCTCCGCCCCGTCGACGGCCTCACCTCCTCGACCCTGC CCGACGTCCTCATCCCGCGCCACCCGGTACTCGCCTTGCACCTCCACTTGCATTATG CTTATTATTTATTTTTTCACAAGAATATATTATTATCTCAAGCTTATTATTATTATTGA ATTTATATATTTCTATTTCTTGTCTCGCAGCATTATTAATAAATTGTTGCAATGGTCC ATTGCAGGAGAGTATCGACAAGCTGCTCAGGGAACTGTCGGCCGAGCAGATCGGCCA GACCATCACCGACCTGTCGCAGCTCTACACGCGCTACTACACGTCCACCACCGGTGTT GAGGGTAGCGGCTCCGAGACACGCGTCACCGCGCACACGACACGACACTACACACGC ACCACCGCACACGCACACGCACACGCACACGCACACGCACACGCACACGCACCACCA GCGGTTCTCACCGGACGGGGTACCGCAGGTGCGAAGCTGCTGCACAGCAAGTACAGC GAGTTCGCCCGCAACCAGTCCCACATCTCTGTGCACTTCTTCGAGCCCACCTGGC-3’	628	96.49
	R	5’-TTGGATCGAGTTCAAGGAGGGCGAGCGCGCGTGGCTCACCGAGATGCAGATCCAGCAG CTCATCAACGAGAAGGCGCACTTCATGGACGTCACTGACCACCCCGACCTCCGCCCCGTCG ACGGCCTCACCTCCTCGACCCTGCCCGACGTCCTCATCCCGCGCCACCCGGTACTCGCCTTG CACCTCCACTTGCATTATGCTTATTATTTATTTTTTCACAAGAATATATTATTATCTCAAGCT TATTATTATTATTGAATTTATATATTTCTATTTCTTGTCTCGCAGCATTATTAATAAATTGTTG CAATGGTCCATTGCAGGAGAGTATCGACAAGCTGCTCAGGGAACTGTCGGCCGAGCAGATC GGCCAGACCATCACCGACCTGTCGCAGCTCTACACGCGCTACTACACGTCCACCACCGGTGT TGAGGGTAGCGGCTCCGAGACACGCGTCACCGCGCACACGACACGACACTACACACGCACC ACCGCACACGCACACGCACACGCACACGCACACGCACACGCACACGCACCACCAGCGGTTC TCACCGGACGGGGTACCGCAGGTGCGAAGCTGCTGCACAGCAAGTACAGCGAGTCGCCCGC AACCAGTCCCAAAATG-3’	630	96.49
**Shwachman-Bodian-Diamond syndrome** ACA1_142090	F	5’-CATCCGACTCGAGCTCGACCCCCGAGGAGTACTGGGTGGTGGCCAACCCGGGCATGCCCG ACAAGTGGAAGCAGGACCCGTCCACCCCGCTCACCGACGTCCTGCAGTCGTTCGACATCTTC AAGGGACACGGCCAGTCGGGCCAGGCCCCCAAGGCCGACCTTCTGTACATCTCCCTTCTTTC TGTGGTGCGTGTCATCGTGTGGTACAGTGTGTGCGGTGCCTAACGCTGCGGGGGCAACTGGTG CAGGAAGGTGTTCGGGACGACAGACAACGACGCCGTGATCCGAAAGATCCTGTCCATCCA-3’	307	96.25
	R	5’- TCGATCGAAGGAATGAAGGTGAGGTTCACATACAAGCCCGACTCGAGCTCGACCCCCGA GGAGTAGTGGGTGGTGGCCAACCCGGGCATGCCCGACAAGTGGAAGCAGGATCCGTCCACC CCGCTCACCGACGTCCTGCAGTCGTTAGACATATTCAAGGGACACGGCCAGTCGGGCCAGGC CCCCAAGGCCGACCTTATGTACATCTCCCTTCTTTCTGTGGTGCGTGTCATCGTGTGGTACAG TGTGTGCGGTGCCTAACGCTGCGGGGGCAACTGTGCAGGAAGTTTCGACACAAACG-3’	301	94.27
**Cell division control protein 2b** (XM_004353658.1)	F	5’-GGAGACTCATGAGAGCTCGGTGAGGGCACCTATGGCGAGGTATACAAGGCCATCGAT ACCGAGAACCAGCGATTCA-3’	76	95.52
	R	5’-ATGCAAGCCAAACCCAGTCCCCTGCAGCGGTACGACATCAAGGAGAAGCTCGGTGAGG CACCTATGCGAGATACGCC-3’	77	97.22

[a] The DNA sequences were blasted against *A. castellanii* in NCBI database.

[#] The DNA sequences of ATG3, ATG8b, ATG12, ATG16, and 18S rRNA were published by Boonhok and colleagues (Boonhok et al., 2021, 2022).
